# Evaluation of the effectiveness of solutions for clearing neonatal central venous catheters: randomized study*

**DOI:** 10.1590/1980-220X-REEUSP-2025-0122en

**Published:** 2025-11-28

**Authors:** Carolina Mathiolli, Juliane Pagliari Araujo, Keli Regiane Tomeleri da Fonseca Pinto, Rosangela Aparecida Pimenta, Danielle Venturini, Sonia Silva Marcon, Adriana Valongo Zani

**Affiliations:** 1 Universidade Estadual de Londrina, Departamento de Enfermagem, Londrina, PR, Brazil.; 2 Instituto Federal do Paraná, Departamento de Enfermagem, Londrina, PR, Brazil.; 3 Universidade Estadual de Londrina, Departamento de Patologia Aplicada, Análise Clínicas e Toxicológicas, Londrina, PR, Brazil.; 4 Universidade Estadual de Maringá, Departamento de Enfermagem, Maringá, PR, Brazil.

**Keywords:** Catheter Obstruction, Infant, Newborn, Heparin, Ascorbic Acid, Saline Solution

## Abstract

**Objective:**

To evaluate the effectiveness of saline, heparin, and vitamin C solutions in unclogging neonatal central venous catheters occluded by clots.

**Method:**

A randomized, double-blind, *in vitro* experimental study conducted in August 2022 with 90 neonatal central venous catheters occluded with blood that remained in a water bath for 8 hours. The solutions tested were: saline, heparin (50 mg/ml), and vitamin C (25 mg/ml), using a negative pressure technique. The variables of number of attempts and time to unclogging were evaluated in relation to the mean and standard deviation. For variables with abnormal distribution, the Kruskal-Wallis test was used.

**Results:**

There was no statistical significance in relation to the time and number of attempts to unclog the three solutions (p > 0.05), and the maximum time to unclog the catheters was 120 minutes.

**Conclusion:**

The three substances showed similar results. Thus, saline solution is recommended because it is considered safe, with fewer side effects when used in newborns.

## INTRODUCTION

The peripherally inserted central catheter (PICC) is a long, flexible device inserted through peripheral venous puncture reaching the distal portion of the inferior or superior vena cava, depending on the insertion site^1,2)^.

According to Resolution 258 of 2001, nurses are supported by the Federal Nursing Council for the indication, insertion, maintenance, and removal of PICC, provided they have the technical and theoretical qualifications to do so, as this is a highly complex procedure. Given this, nurses are one of the main parties responsible for infection control and the prevention of obstruction of this long-term venous device, especially in the neonatal population^(3)^.

PICC is especially indicated for newborns (NB), as the catheter is made of low thrombogenicity materials (polyurethane and silicone), providing prolonged and safe intravenous therapy, in addition to reducing the number of punctures and, consequently, painful procedures^4,5)^. For the neonatal population, the main indications for choosing this device are related to the need for intravenous therapy for six days or more, infusion of hyperosmolar solutions, sedatives, vasoactive drugs, total or partial parenteral nutrition, chemotherapy drugs, and vesicant and/or irritant medications^(6)^.

Despite the benefits, the use of this device can lead to complications, which can impair the health of the newborn. The rates of complications range from 32.8 to 48.8%, with obstruction being the most frequent (5.55 to 21.40%) and infection (1.85 to 14.30%), rupture (1.1 to 13.04%), infiltration (4.34 to 12.03%), and accidental removal (2.4 to 11.11%)^7)^.

Among the complications, catheter obstruction stands out. This research focuses on obstruction by clots that occur due to their presence inside the lumen or at the distal end, which can cause partial or total occlusion. It is evidenced by difficulty in infusing medications or aspirating blood through the device. The incidence of obstruction in newborns ranges from 9.7% to 30.9%^8,9)^.

Prevention of occlusion is considered the best care strategy for central venous catheters, which can be ensured by flushing with saline solution before and after infusions of substances. However, some literature indicates the continuous infusion of 0.5 U/kg of heparin in saline solution^10)^. In contrast, a systematic review presented in its results the absence of scientific evidence to affirm that there is a difference between flushing with heparin and flushing with saline solution for maintaining central venous access in newborns and children^11)^. Corroborating this research, a randomized clinical trial demonstrated that there was no statistical difference between flushing with vitamin C and flushing with saline in preventing central venous catheter obstruction in children^12)^. In addition, authors state that there is a lack of studies establishing the safety, efficacy, and efficiency of solutions^(10)^.

Although there is a lack of scientific evidence for the prevention of obstruction and restoration of PICC patency, it is known that in daily practice, various types of solutions are used to unclog central venous catheters in order to recover these devices^13,14)^due to the impossibility of new central venous access as a result of hemodynamic instability and/or venous network difficulty.

Given this, the research question was formulated according to the PICO mnemonic strategy (Population (P), Intervention (I), Comparison (C), and Outcome (O)) where P: peripheral insertion central venous catheter in newborns occluded by clots, I: infusion of saline solution, C: infusion of heparin and infusion of vitamin C, and O: catheter unclogging. “How effective is saline solution compared to heparin and vitamin C in unclogging peripheral insertion central venous catheters in newborns occluded by clots?”

This study hypothesis follows: Is there a difference in the effectiveness of saline solution, heparin, and vitamin C in unclogging neonatal peripherally inserted central venous catheters occluded by clots? Therefore, the objective was to evaluate the efficacy of saline, heparin, and vitamin C solutions in clearing peripheral insertion central venous catheters in newborns occluded by clots.

## METHOD

### Type of Study

This is a double-blind, randomized, in vitro experimental study. In vitro experimental studies consist of conducting an experiment in a controlled laboratory environment to obtain data on the safety and efficacy of a procedure under test, given the impossibility of performing it on living individuals^(15)^.

Considering that the neonatal unit has a population of critically ill patients, the interventions performed should not further impair their prognosis. Therefore, in order to establish the minimum necessary doses of the substances used to unblock venous access, it would be inappropriate to initially conduct the experiment in vivo. In addition, evaluation of the device in an extracorporeal environment with the naked eye allows the detection of possible damage to the integrity of the venous catheter and the detachment of clots visible to the distal part of the PICC.

### Study Site

The study was conducted in a postgraduate laboratory belonging to a state university in Paraná. The site was used exclusively for data collection. The catheters were collected in the intensive care and intermediate care neonatal unit of the university hospital.

### Population and Sample

The sample consisted of 90 catheters used by newborns hospitalized in a neonatal unit. These devices received parenteral nutrition or antibiotic therapy or a glucose solution with a glucose infusion concentration above 12 or vasoactive drugs.

After the end of treatment, the venous devices were removed and evaluated for integrity and the presence of kinks and twists, and then washed with 2 ml of distilled water in a 10 ml syringe to ensure their permeability. In addition, they were placed in individual plastic bags and stored in an opaque box protected from light and moisture. To identify the catheters used in the study, they were labeled with the following information: dates of insertion and removal, reason for removal, therapy infused, and length in centimeters.

### Selection Criteria

The inclusion criteria were newborn catheters with a 2*French*gauge; polyurethane material; belonging to the same manufacturer; pervious; minimum length of 11 centimeters; having been kept in the newborn for at least seven and at most 30 days; having been stored for a maximum of two years; and having all characterization data on their identification labels. It took two years to reach the number of catheters needed to compose the sample size. Catheters that were not stored properly and/or were not patent and/or were not intact and/or had folds and/or twists at the time of separation for the study were excluded.

It was necessary for the stored catheters to be patent, as the study aimed to simulate, as closely as possible, the unblocking technique used in neonatal units. In practice, the obstruction is identified and attempts to unblock it are initiated immediately. As this was an experimental study, the same environmental conditions were provided for the solutions in the attempt to unblock the PICC.

### Calculation of Sample Size

To calculate the sample size, the electronic medical records of the study’s reference neonatal unit were used to collect information on the number of PICC removed due to obstruction or end of treatment in the last year, from May 1, 2021, to May 31, 2022, considering that a pilot study had been conducted previously in June. A total of 121 PICC lines were removed, resulting in a sample size of 82 catheters for all three groups. It should be noted that a 5% sampling error and a 95% confidence level were considered. Although the sample calculation referred to 82 catheters as a safety margin, the researchers opted to establish a higher number, i.e., 90 catheters were used.

### Data Collection

Prior to data collection, a pilot study was conducted in June 2022 following the same criteria adopted for data collection, with 10% of the total sample size, i.e., three catheters in each of the three groups, totaling nine PICC.

In the pilot study, it was possible to determine the time required for catheter obstruction to occur (8 hours). The same techniques used in the intervention were employed for unclogging attempts. However, it was noticed in the pilot test that the 10 ml*Luer-Lock*syringe provided greater negative pressure and fewer disconnections when compared to the 10 ml*Luer-Slip*syringe, therefore, the use of the 10 ml *Luer-Lock*syringe was standardized in the intervention.

The temperature and humidity of the laboratory were controlled using an ambient thermometer, at one-hour intervals, throughout the six days of collection. The temperature ranged from 21.1°C to 25.1°C, and the humidity varied from 37% to 56%.

Prior to the start of the intervention, all devices were retested with a 10 ml syringe and distilled water to confirm their permeability, and their integrity and the presence of folds and twists were also assessed—those in which these alterations were present were discarded. In addition, the information on the catheter labels was rechecked and transferred to a spreadsheet. Then, all catheters were cut to 11 cm using a scalpel blade to standardize the devices, since in practice, a length of 5 to 30 cm is used for insertion. The devices were then numbered and marked with a permanent pen from 1 to 102 at random. All 102 PICC were allocated into groups according to the length of stay of the newborn, storage time, and infused therapy. The length in centimeters of storage was not considered, as the catheters were all cut to 11 centimeters. It should be noted that 12 more catheters than the total sample were included to ensure that no damage occurred during collection.

Data collection was performed in August 2022 on six different intervention days. Each day, 17 catheters were randomly selected, ensuring that the intervention groups were as homogeneous as possible according to the length of stay in the NICU, storage time, and infused therapy. Seventeen catheters were selected to ensure the obstruction of at least 15 devices per day, distributed equally among three groups. Blood was aspirated from the same volunteer donor for each PICC to cause its obstruction.

For donor selection, it was determined that the donor should not be undergoing any type of health treatment and should not have any changes in coagulation factors. The results of the tests collected were: blood type A+, activated partial thromboplastin time of 34.3 seconds and P/N ratio of 1.11; prothrombin time of 11.1 seconds; activity of 116%; P/N ratio of 0.94; platelets of 370.000/µL; hemoglobin of 13.6 g/dL; red blood cells of 4.42 million/µL; hematocrit of 40.1%; total leukocytes of 7720 µ/L. It was decided to use blood from an adult donor so that there would be no damage resulting from puncture for blood collection from a newborn severely hospitalized in the neonatal unit.

Each day, 3 ml of blood was collected from the same donor by venipuncture using a #23 Scalp needle and a 10 ml syringe. The blood was placed in an open container, into which the distal end of the catheter was inserted to aspirate 0.05 ml (priming volume) of blood from the 17 catheters using a 1 ml syringe. The time for blood collection and aspiration was approximately two minutes.

Next, using Kelly clamps, the distal end of the catheters was clamped, and the proximal end was occluded one by one with 1 ml syringes to prevent the entry of air and/or distilled water and/or the outflow of blood from the PICC lumen.

The catheters were submerged in water bath with distilled water at 37°C (Quimis Aparelhos Científicos Ltda.), where they remained for 8 hours using the*overnight*technique. The temperature of 37°C was chosen to simulate the normothermic temperature of newborns.

To ensure thermal control during the night, temperature tests were performed with the device before data collection. The temperature of the distilled water ranged from 36.0°C to 38.0°C. To ensure that the catheters did not touch the bottom of the device and cause the PICC to overheat, a kidney dish was placed inside the device to protect them.

Over the six days, the venous devices were placed in a water bath between 12:00 a.m. and 12:30 a.m. and remained there until 8:00 a.m. to 8:30 a.m.

After eight hours in the water bath, one centimeter of the distal end of the catheters, which had been occluded with Kelly clamps, was cut with a scalpel to ensure the integrity of the device. Thus, in the end, all PICC were 10 cm long. Next, the occlusion was tested by attempting to infuse saline solution into a 10 ml syringe. The occlusion was verified by the resistance and inability to infuse the saline solution. The catheter occlusion technique was based on two previous studies^16,17)^.

**Figure 1:**
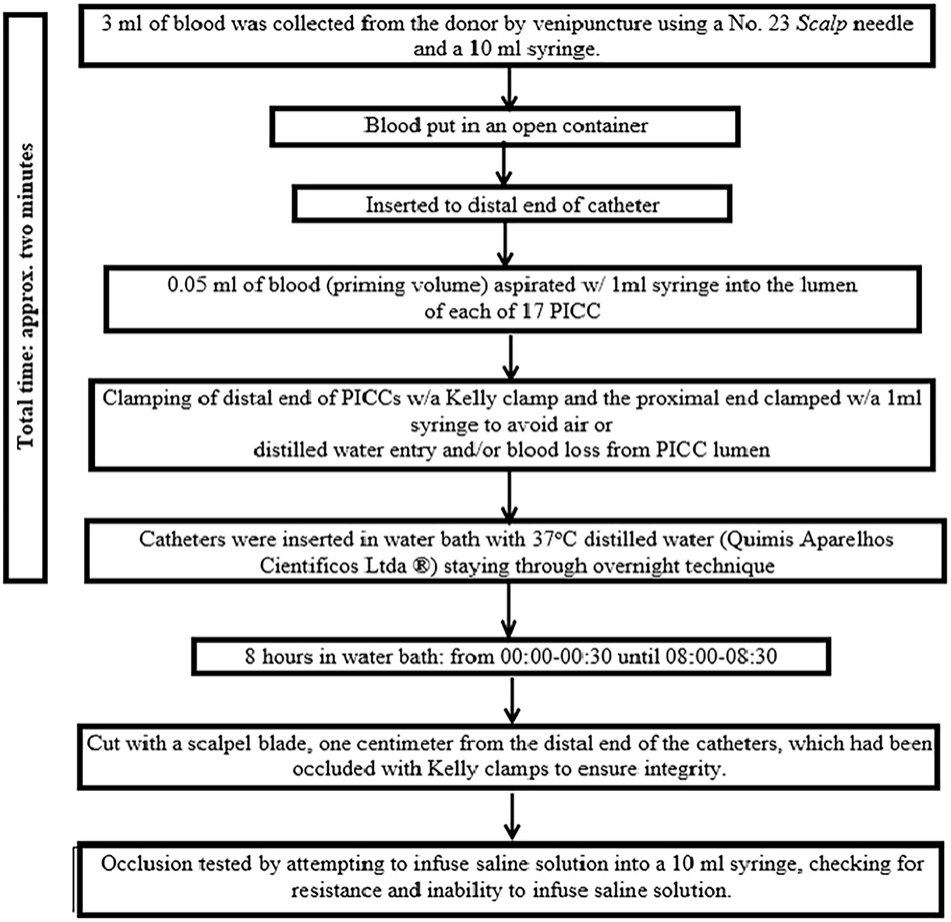
Description of the method of obstructing central venous catheters. Londrina, Paraná, 2022.

A flowchart was developed describing the method of obstructing venous catheters with blood, as shown in Figure 1.

Authors of the study, 2022.

Catheters that did not become obstructed were classified as losses, and the first 15 obstructed catheters were included in the study. If there were more than 15 obstructed PICC, they were discarded as losses so that each group would receive five catheters equally each day. The study showed four catheters that did not occlude, and eight were discarded to ensure homogeneity between the groups.

The first 15 occluded devices were randomly distributed by lottery into three groups. Each group received a different solution to test its unclogging ability, and the distribution of group numbers was random by lottery.

Group 1 received a 10 ml syringe filled only with saline solution.

Group 2 received injectable vitamin C at a concentration of 25 mg/ml diluted in saline solution in a 10 ml syringe. Due to the lack of studies on the use of injectable vitamin C in clearing catheters in newborns, the concentration of the solution was calculated based on the recommended daily dose for premature babies, which is 15–25 mg/kg/day^18)^.

Group 3 received low molecular weight heparin at a dose of 50 mg/ml diluted in saline solution in a 10 ml syringe. The dose of low molecular weight heparin was determined through a previous*in vitro*experimental study that determined that low molecular weight heparin at a concentration of 50 mg/ml was effective in unclogging PICC lines in newborns^(16)^.

The syringes containing the solutions were protected from light using laminated paper and labeled with colored tape, the color of which was determined by lottery to differentiate the solution being tested. Saline solution, group 1, received green tape; vitamin C, group 2, received yellow tape; and heparin, group 3, received red tape.

The technique adopted for PICC unclogging was negative pressure using a three-way stopcock, with the clogged catheter connected to one channel, a 10 ml empty Luer-Lock syringe was placed at the proximal end of the tap, and a 10 ml Luer-Lock syringe with the test solution was placed at the distal end of the three-way tap^(19)^.

In this technique, the solution line is closed and the contents of the catheter lumen are aspirated up to the 10 ml mark on the empty syringe; then, the empty syringe line is closed and the syringe line with the solution is opened, allowing the solution to fill the catheter through the negative pressure generated in the PICC lumen. At no time is the infusion technique performed, only aspiration, thus aiming to aspirate clots and solution^(19)^.

The attempt to unblock the catheter was performed with the catheters submerged in a water bath of distilled water at 37°C for 1, 5, 10, 15, 30, 60, 120, and 240 minutes, with three attempts at unblocking performed at each time interval. These time intervals were determined for the best analysis of arithmetic progression. Every 240 minutes, if the PICC permeability was not restored, a new solution was prepared due to the instability of heparin. The maximum unclogging time was determined to be 24 hours after the occlusion was identified.

The recovery of venous catheter patency was considered when the aspiration of clots and distilled water from the water bath in which they were submerged was observed. To confirm the unclogging, 1 ml of saline solution was infused into a graduated device in one minute. After the start of data collection, no changes to the study protocol were necessary.

### Randomization

**Figure 2:**
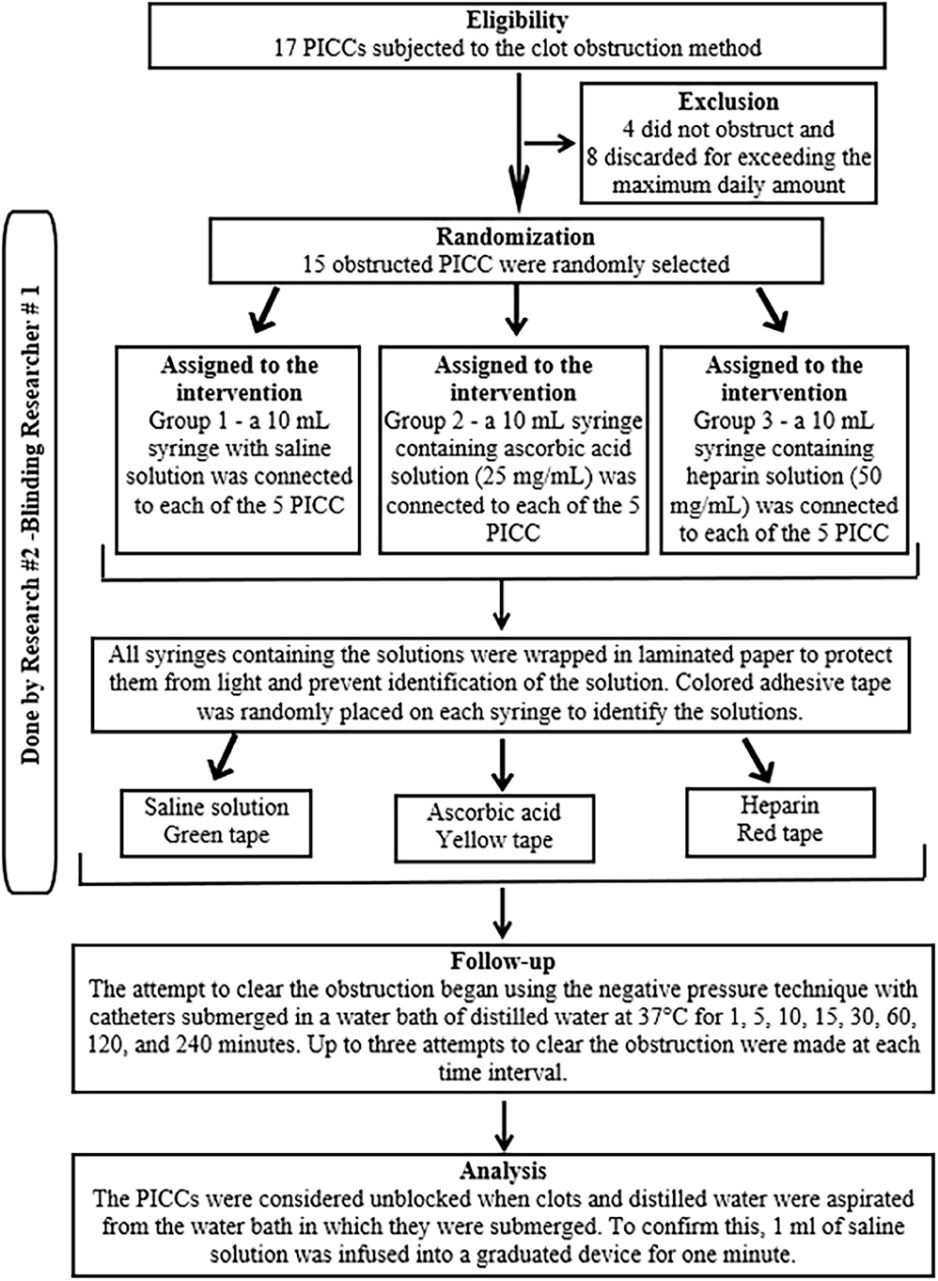
Description of the randomization of blocked central venous catheters for the start of unblocking attempts. Londrina, Paraná, 2022.

Simple randomization was performed by a professional external to the research, responsible for preparing three opaque envelopes, identified with green, blue, and yellow ribbons, which determined which group each solution belonged to. This same professional also identified the syringes with the respective colors and corresponding solutions. The order of selection of the PICC for the attempt at unblocking was determined by simple randomization, as shown in the flowchart in Figure 2.

Authors of the study, 2022.

### Blinding

Two researchers participated in data collection, with researcher 1 attempting to unblock all catheters over the six days and researcher 2 responsible for randomizing the solutions to be tested in each catheter. Blinding was performed by researcher 1, who performed the negative pressure technique to unblock the PICC, and by the person responsible for performing the statistical analysis of the data.

The catheter number, solution number (1, 2, 3), number of attempts, and unclogging time were recorded in a spreadsheet. Data were double-entered and double-checked.

### Data Analysis and Treatment

The tests were performed using the Statistical Package for the Social Sciences (SPSS (25.0) program). Descriptive analysis was presented using mean and standard deviation (SD). The variables of number of attempts and time to unblocking were evaluated using data normality analysis through the Shapiro-Wilk test. The variables presented abnormal distribution; therefore, the difference between the intervention groups was evaluated using the Kruskal-Wallis test. A significance level of 5.0% and p ≤ 0.05 were adopted.

### Ethical Aspects

This study is part of a research project entitled “Barreiras e potencialidades no cuidado com o cateter central de inserção periférica em neonatologia: buscando inovações”. (Barriers and potentialities in the care of peripherally inserted central catheters in neonatology: seeking innovations.) The research was cleared by the Research Ethics Committee, under No. CEP/UEL: 4.324.095. CAAE: 38696720.9.0000.5231 and was conducted in accordance with the necessary ethical precepts.

The need for approval from the parents and/or guardians of the newborns through the signing of the free and informed consent form (FICF) for the use of the PICC in their children was waived, as the venous device would be discarded, since its removal occurred after the end of treatment. In addition, because a flush was performed with 2 ml of distilled water, the catheters did not contain any biological material from the patient.

## RESULTS

Table 1 was prepared, containing the characterization of the 90 PICC that made up the sample. It can be observed that the most frequent catheter storage length was 16 to 20 cm, most catheters were stored for a period of 0 to 6 months, 7 to 12 days was the most prevalent length of stay in the NICU, most devices received antibiotic therapy infusion, and all catheters were removed due to the end of treatment.

**Table 1:** Characterization of the PICC catheters that comprised the sample – Londrina, PR, Brazil, 2022.

Characteristics	Catheters in the sample (n = 90)
Length of catheter in storage	24.45% (11–15 cm)
	31.12% (16–20 cm)
	27.78% (21–25 cm)
	16.65% (26–30 cm)
Time of catheter’s storage	31.12% (0–6 months)
	18.88% (7–12 months)
	27.77% (13–18 months)
	22.23% (19–24 months)
Time of permanence in NB	62.06% (7–12 days)
	24.14% (13–18 days)
	6.90% (19–24 days)
	6.90% (25–30 days)
Type of infused therapy	60.65% (antibiotic therapy)
	31.14% (parenteral nutrition)
	4.92% (Vasoactive drug)
	3.29% (glucose solution with a glucose infusion concentration above 12)
Reason for withdrawal	100% (end of treatment)

Source: Research data.

Table 2 shows that the means and standard deviations for attempts at clearing the obstruction were 8.47 (5.23) for saline solution (substance 1), 9.43 (5.61) for vitamin C (substance 2), 8.20 (5.59) for heparin (substance 3), and a p-value of 0.639 according to the Kruskal-Wallis test. In relation to the clearing time in minutes, the means and standard deviation were 30.80 (40.63) for saline solution (substance 1), 35.43 (45.68) for vitamin C (substance 2), 29.17 (41.17) for heparin (substance 3), and a p-value of 0.758 by*Kruskal-Wallis.*

**Table 2:** Evaluation of the difference between the intervention groups associated with the number of attempts and the time in minutes – Londrina, PR, Brazil, 2022.

Variables, X* (SD)**	Total	Solutions			p-value^†^
		1^††^	2^†††^	3^††††^	
Clearing attempts	8.70 (5.45)	8.47 (5.23)	9.43 (5.61)	8.20 (5.59)	0.639
Time(minutes)	31.80 (42.16)	30.80 (40.63)	35.43 (45.68)	29.17 (41.17)	0.758

X*: mean.

SD**: standard deviation.

^†^Kruskal-Wallis test.

1^††^: saline solution.

2^†††^: vitamin C.

3^††††^: heparin.

Source: Research data.

Table 3 presents a description of the solutions for unclogging PICC in relation to time intervals and attempts. It can be observed that the number of catheters unclogged by each of the three solutions was similar throughout each time period (1, 5, 10, 15, 30, 60, and 120 minutes). The maximum time for clearing the catheters using the three solutions was 120 minutes, but the second and third attempts at clearing were not necessary within 120 minutes for the saline solution, unlike the vitamin C and heparin solutions.

**Table 3:** Unclogging of occluded catheters in the laboratory according to the three solutions related to time and attempts – Londrina, PR, Brazil, 2022.

Time	Attempt	Solution 1 (saline solution)	Solution 2 (vitamin C)	Solution 3 (heparin)
		Number of unclogged catheters (n)		
1 minute	1ª	1	0	2
	2ª	2	2	2
	3ª	6	6	6
Subtotal		9/30 (30%)	8/30 (26.66%)	10/30 (33.33%)
5 minutes	1ª	1	0	1
	2ª	2	2	3
	3ª	1	0	1
Subtotal		13/30 (43.33%)	10/30 (33.33%)	15/30 (50%)
10 minutes	1ª	2	2	0
	2ª	1	2	1
	3ª	1	3	2
Subtotal		17/30 (56.66%)	17/30 (56.66%)	18/30 (60%)
15 minutes	1ª	0	1	0
	2ª	0	0	1
	3ª	1	0	1
Subtotal		18/30 (60%)	18/30 (60%)	20/30 (66.66%)
30 minutes	1ª	0	1	0
	2ª	1	0	1
	3ª	3	3	1
Subtotal		22/30 (73.33%)	22/30 (73.33%)	22/30 (73.33%)
60 minutes	1ª	1	0	1
	2ª	1	0	2
	3ª	2	2	1
Subtotal		26/30 (86.66%)	24/30 (80%)	26/30 (86.66%)
120 minutes	1ª	4	0	1
	2ª	–	2	1
	3ª	–	4	2
Total		30/30 (100%)	30/30 (100%)	30/30 (100%)

Source: Research data.

During attempts to clear the blockage, no visible damage to the integrity of the PICC or detachment of clots visible to its distal part was observed with the naked eye, but it was possible to visualize the aspiration of clots into the syringe in which negative pressure was applied.

## DISCUSSION

When evaluating the effectiveness of saline, heparin, and vitamin C solutions in unclogging neonatal central venous catheters occluded by clots, it was found that all three substances were effective in unclogging catheters by clots.

Clot obstruction within the PICC lumen can occur partially or totally due to the presence of fibrin or blood clots, making it difficult or impossible to administer medications due to the lack of permeability. It is usually associated with retrograde flow within the catheter or failure to perform flushing^20)^. Thrombus is one of the main causes of central venous device obstruction. Thrombus formation occurs due to the so-called Virchow triad: blood stasis, endothelial injury, and hypercoagulability^(21)^.

This study demonstrated that saline solution was as effective as heparin at a concentration of 50 mg/ml and vitamin C at a concentration of 25 mg/ml for clearing PICC lines clogged with blood*in vitro*using the negative pressure technique.

Saline solution is composed of an aqueous solution with a 0.9% concentration of sodium chloride and can be used intravenously, intraocularly, inhalationally, among others. It is a compound with high microbiological and physicochemical stability^22)^. Among crystalloids, 0.9% saline solution is the most widely used, despite having high concentrations of chlorine and sodium in relation to plasma. It has higher osmolality, but close to that of blood. After infusion of this solution, chlorine and sodium remain in the extracellular fluid, which allows saline solution to be considered an isotonic solution^23)^. The anticoagulant effects of saline solution are unknown.

Vitamin C is an essential micronutrient that is part of various biological and biochemical processes. There are two forms of vitamin C in the blood: dehydroascorbate (oxidized form) and ascorbic acid. This vitamin acts by blocking platelet adhesion to the vascular endothelium and through platelet aggregation mediated by P-selectin and induced by thrombin^(24)^.

Corroborating the findings of this study, randomized trials comparing vitamin C and saline have shown that there is no significant difference between these two solutions in preventing central venous catheter occlusion^(12,25)^.

In the presence of small clots, the infusion of ascorbic acid unblocks the blood vessel and removes platelet adhesion to the endothelium. In addition, vitamin C acts by inhibiting the release of thrombin, which depends on the pH of platelet plasminogen activator inhibitor-1. It also reduces the concentrations of von Willebrand factor and tissue plasminogen activator, demonstrating an important role in coagulation and inflammation^24)^. There is a lack of scientific evidence addressing the adverse effects of vitamin C in the neonatal population.

Heparin is formed by polysaccharide chains of various molecular weights and has a high affinity for the physiological inhibitors of coagulation, antithrombin, activated factor X (Xa), and thrombin (IIa). In this study, low molecular weight heparin was used. Its anticoagulant function is performed by antithrombin III, which acts by inhibiting coagulation factors IXa, XIa, to a lesser extent, and XIIa and IIa and Xa to a greater extent^26)^. Enoxaparin is the most widely used low molecular weight heparin, as it requires a lower dose for Xa to reach adult values and has a lower clearance in neonates. Among the adverse effects, bleeding stands out^(27)^.

Corroborating the findings of this study, randomized studies have analyzed that heparin and saline solution showed no statistically significant difference in terms of efficacy in maintaining the patency of central venous devices; therefore, the use of saline solution was recommended^28,29)^.

Regarding the unblocking of venous devices obstructed by clots, the use of heparin is recommended, especially in the national scenario^16)^, and alteplase, especially in the international scenario^14)^. Until 1999, the United States recommended the use of urokinase, but the Food and Drug Administration (FDA) banned the use of this drug due to risks to its safety and efficacy, as it was associated with severe bleeding events^14,30)^.

It is important to consider that in sick newborns, there is a lack of control of the levels of these coagulation factors, which may be related to several different causes, such as a procoagulant state, endothelial damage, and reduced flow, favoring thrombus formation or bleeding^(27)^.

This study used the negative pressure technique; however, in the literature, there are two techniques for unblocking central catheters: the infusion of solutions and the negative pressure technique^(31)^.

When partial occlusion of the PICC occurs, detected by increased resistance in the administration of medications, the technique of infusing the medication with the solution is recommended to restore permeability. In the case of total occlusion of the PICC, the negative pressure technique should be used to minimize the risk of damage^(31)^.

As obstruction can occur in any internal segment of the PICC, it is possible that the catheter has solution between the obstruction and the proximal end, so the negative pressure technique aims to remove this solution and cause intraluminal negative pressure in the venous device. When the negative pressure is released, the substance present in the other syringe connected to the catheter via the three-way stopcock is aspirated^(32)^.

This technique reduces the damage that overpressurization can cause to the PICC and allows the solution responsible for unclogging the catheter to reach the thrombus causing the occlusion more quickly, as it is not necessary to force the syringe plunger for the solution to enter the intraluminal region of the venous device^(32)^.

The catheter should be manipulated with a syringe of at least 10 ml so as not to cause pressure greater than 25 psi inside the catheter, avoiding catheter rupture and damage to the venous network^(33)^.

It is known that obstruction of a venous catheter has a number of consequences and negative impacts for the newborn and their family, including cessation of drug therapy, increased hospital costs, stress, increased painful procedures, and repeated insertions for a new venous device^34)^. It should be noted that PICC in newborns has a higher chance of occlusion due to its small caliber^(12)^.

Randomized clinical trials show a lack of scientific evidence to support the recommendation to use heparin^35)^, saline solution, and ascorbic acid^12)^to maintain the patency of central venous catheters. This corroborates the findings of this study that there was no statistically significant difference between the solutions for unclogging PICC catheters.

Several strategies have been developed to prevent venous catheter obstruction, such as the creation of an anti-reflux valve that is placed at the distal end of the PICC to prevent blood reflux into the catheter lumen. However, scientific studies have not demonstrated a reduction in the rate of occlusion, and, on the contrary, it is a strategy that increases the cost of the venous device^(36)^.

Regarding venous catheter care, the nursing team stands out, especially the nurse, who is the professional responsible for defining and performing the most appropriate dressing for the patient, in addition to performing measures to prevent infection and obstruction. It is the nurse’s responsibility to ensure proper handling, evaluate venous devices daily, and train the health team responsible for newborn care^(37)^.

Among the care measures to be taken to prevent occlusion is the instillation of saline solution with positive pressure to prevent blood from returning into the catheter lumen^19)^, which should be done every six hours or before and after the infusion of medications. For this, it is necessary to infuse saline solution using the pulsatile technique in order to cause turbulence inside the catheter so that fibrin deposits are removed^(12)^.

It is considered a limitation that no microscopic analyses of the inside of the catheters were performed to ascertain partial or total unblocking, thus verification of unblocking was done only by clinical observation, that is, the catheter was considered unblocked when saline solution was instilled in the proximal region and fluid was observed in its distal part without resistance to infusion. Another limiting factor was the two years of collection and storage required to obtain a sufficient number of PICC to reach the sample size, since during this time, in the neonatal unit to which this research is linked, there were catheters that were removed due to obstruction and rupture and therefore could not be included in this research.

In vitro studies are essential for investigating biological mechanisms and initially testing the efficacy and safety of procedures in a controlled environment. However, the validity of these studies for direct application in humans, especially in neonatology, is limited, as they do not fully reproduce the complexity of the living organism, including immune responses, metabolism, and systemic cellular interactions. Therefore, in vitro findings should be interpreted as a preliminary step that needs confirmation through in vivo and clinical studies, ensuring real safety and efficacy for neonatal patients^(38–40)^.

In addition, direct extrapolation of in vitro results to clinical practice requires caution, as factors such as the physiological environment, individual variability, and specific clinical conditions of newborns can influence the behavior of the device or substance being tested. Therefore, for interventions based on *in vitro* data to be adopted in neonatal nursing care, it is essential to conduct rigorous clinical research to validate these findings and promote evidence-based practice, ensuring the quality and safety of care^38,39)^.

It is hoped that this research will serve as a basis for future studies exploring safer and more effective alternatives for PICC line clearing in newborns, especially with regard to comparing different solutions and techniques used in clinical practice. Among the main gaps to be addressed in future research are the evaluation of long-term adverse effects, the standardization of management protocols, and the cost-benefit analysis of interventions. From a practical point of view, the results of this study can support nursing care, promoting safer and evidence-based practices, which contributes to reducing complications and improving clinical outcomes for newborns. It is also important to emphasize the importance of adopting health practices based on scientific evidence, ensuring qualified care focused on patients’ needs.

## CONCLUSION

The three substances used were effective in clearing clots from the catheters, as all catheters had their patency restored within a maximum of 120 minutes using the negative pressure technique. This time of up to 120 minutes is feasible in clinical practice, since the protocol for unclogging central venous catheters in the neonatal unit linked to this study considers a maximum time of 24 hours for the recovery of the permeability of these devices.

Thus, the hypothesis of this study was refuted since there was no statistical significance between the solutions used to unclog PICC lines in relation to time and number of attempts.

The use of saline solution is recommended, as it has physical- chemical and microbial stability, causing fewer side effects in relation to its use in newborns, especially when compared to other solutions.

## Data Availability

The entire dataset supporting the results of this study was published in the article itself.
