# Supplementary material for: Evaluation of the effectiveness of solutions for clearing neonatal central venous catheters: randomized study
Source: Rev Esc Enferm USP. 2025 Nov 28;59:e20250122. doi: 10.1590/1980-220X-REEUSP-2025-0122en (PMC12662237; doi:10.1590/1980-220X-REEUSP-2025-0122en)
Supplement: Translation

# Translation: Avaliação da eficácia de soluções para desobstrução de cateter venoso central neonatal: estudo randomizado*

**DOI:** 10.1590/1980-220X-REEUSP-2025-0122pt

**Authors:** Carolina Mathiolli, Juliane Pagliari Araujo, Keli Regiane Tomeleri da Fonseca Pinto, Rosangela Aparecida Pimenta, Danielle Venturini, Sonia Silva Marcon, Adriana Valongo Zani

**Affiliations:** 1 Universidade Estadual de Londrina, Departamento de Enfermagem, Londrina, PR, Brasil.; 2 Instituto Federal do Paraná, Departamento de Enfermagem, Londrina, PR, Brasil.; 3 Universidade Estadual de Londrina, Departamento de Patologia Aplicada, Análise Clínicas e Toxicológicas, Londrina, PR, Brasil.; 4 Universidade Estadual de Maringá, Departamento de Enfermagem, Maringá, PR, Brasil.

**Keywords:** Obstrução do Cateter, Recém-Nascido, Heparina, Ácido Ascórbico, Solução Salina

## Abstract

**Objetivo:**

Avaliar a eficácia das soluções de soro fisiológico, heparina e vitamina C na desobstrução de cateter venoso central neonatal ocluído por coágulos.

**Método:**

Estudo experimental duplo-cego randomizado *in vitro* realizado em agosto de 2022 com 90 cateteres venosos centrais neonatais ocluídos com o sangue que permaneceram em banho-maria por 8 horas. As soluções testadas foram: soro fisiológico, heparina (50 mg/mL) e vitamina C (25 mg/mL), por técnica da pressão negativa. As variáveis do número de tentativas e tempo para desobstrução foram avaliadas em relação à média e desvio padrão. Para variáveis com distribuição anormal utilizou-se o Teste de Kruskal-Wallis.

**Resultados:**

Não houve significância estatística em relação ao tempo e o número de tentativas de desobstrução das três soluções (p > 0,05) e o tempo máximo de desobstrução dos cateteres foi de 120 minutos.

**Conclusão:**

As três substâncias apresentaram resultados similares. Deste modo, recomenda-se a solução salina por ser considerada segura, trazendo menores efeitos colaterais em relação ao seu uso em recém-nascidos.

## INTRODUÇÃO

O cateter central de inserção periférica (*peripherally inserted central catheter* – PICC) é um dispositivo longo e flexível inserido através da punção venosa periférica atingindo até a porção distal da veia cava inferior ou superior, de acordo com o local de inserção^(1,2)^.

De acordo com a Resolução 258 de 2001, o enfermeiro possui respaldo do Conselho Federal de Enfermagem para indicação, inserção, manutenção e retirada do PICC, desde que possua habilitação técnica e teórica para tal, pois se trata de um procedimento de alta complexidade. Frente a isso, o enfermeiro é um dos principais responsáveis no controle de infecção e na prevenção de obstrução desse dispositivo venoso de longa permanência, especialmente na população neonatal^(3)^.

O PICC é indicado, especialmente, para recém-nascidos (RN), pois o cateter é constituído por materiais de baixa trombogenicidade (poliuretano e silicone), por proporcionar um tempo de terapia intravenosa prolongada e segura, além de reduzir o número de punções e, consequentemente, procedimentos dolorosos^(4,5)^. Para a população neonatal as principais indicações para escolha deste dispositivo estão relacionadas a necessidade de terapia intravenosa por seis dias ou mais, infusão de soluções hiperosmolares, sedativos, drogas vasoativas, nutrição parenteral total ou parcial, quimioterápicos e medicações vesicantes e/ou irritantes^(6)^.

Apesar dos benefícios, o uso desse dispositivo pode acarretar em complicações, vindo a prejudicar o estado de saúde do RN. As taxas de ocorrência de complicações variam de 32,8 a 48,8%, sendo a obstrução a mais frequente (5,55 a 21,40%) e, com menores percentuais, a infecção (1,85 a 14,30%), ruptura (1,1 a 13,04%), infiltração (4,34 a 12,03%) e remoção acidental (2,4 a 11,11%)^(7)^.

Dentre as complicações, destaca-se a obstrução do cateter. Enfoca-se nesta pesquisa a obstrução por coágulos, que acontece devido a sua presença no interior do lúmen ou na extremidade distal, podendo ocluir de forma parcial ou total. É evidenciada pela dificuldade em infundir medicamentos ou aspirar sangue pelo dispositivo. A incidência de obstrução no RN varia de 9,7% a 30,9%^(8,9)^.

A prevenção da oclusão é considerada a melhor estratégia de cuidado para o cateter venoso central que pode ser garantida pela realização de *flushing* de soro fisiológico antes e após as infusões de substâncias. No entanto, algumas literaturas indicam a infusão contínua de 0,5 U/kg de heparina em solução salina^(10)^. Em contrapartida, uma revisão sistemática apresentou em seus resultados a ausência de evidências científicas para afirmar que há diferença do *flushing* com heparina e do *flushing* com soro fisiológico para a manutenção do acesso venoso central em RN e crianças^(11)^. Corroborando essa pesquisa, um ensaio clínico randomizado demonstrou que não houve diferença estatística entre o *flushing* com vitamina C e o *flushing* com soro fisiológico na prevenção da obstrução de cateter venoso central em crianças^(12)^. Além disso, autores afirmam que faltam estudos que estabeleçam a segurança, eficácia e eficiência de soluções^(10)^.

Embora faltem evidências científicas para a prevenção da obstrução e para a restauração da permeabilidade do PICC, sabe-se que na prática diária são utilizados vários tipos de soluções para a desobstrução de cateteres venosos centrais com o objetivo de recuperar estes dispositivos^(13,14)^ devido a impossibilidade de um novo acesso venoso central em decorrência da instabilidade hemodinâmica e/ou dificuldade de rede venosa.

Diante disso, foi elaborada a pergunta de pesquisa de acordo com a estratégia mnemônica PICO (População (P), Intervenção (I), Comparação (C) e Desfecho (O)), sendo P: cateter venoso central de inserção periférica de recém-nascido ocluído por coágulos, I: infusão de soro fisiológico, C: infusão de heparina e infusão de vitamina C e O: desobstrução do cateter. “Qual a eficácia do soro fisiológico em comparação à heparina e à vitamina C na desobstrução de cateter venoso central de inserção periférica de recém-nascido ocluído por coágulos?”

Este estudo tem como hipótese: Há diferença na eficácia entre o soro fisiológico, a heparina e a vitamina C na desobstrução de cateteres venosos centrais de inserção periférica neonatal ocluídos por coágulos? Portanto, o objetivo foi avaliar a eficácia das soluções de soro fisiológico, heparina e vitamina C na desobstrução de cateter venoso central de inserção de periférica de recém-nascido ocluído por coágulos.

## MÉTODO

### Tipo do Estudo

Trata-se de um estudo experimental duplo-cego randomizado *in vitro*. Estudos experimentais *in vitro* consistem na realização de um experimento desenvolvido em um ambiente laboratorial controlado, a fim de obter dados sobre a segurança e a eficácia de um procedimento em teste, diante da impossibilidade de realização em indivíduos vivos^(15)^.

Considerando que na unidade neonatal encontra-se uma população de pacientes graves, as intervenções realizadas não devem prejudicar ainda mais o prognóstico destes. Portanto, para que sejam estabelecidas as doses mínimas necessárias das substâncias empregadas na desobstrução dos acessos venosos, seria inadequado, inicialmente, que a experimentação fosse feita *in vivo*. Além disso, a avaliação do dispositivo em ambiente extracorpóreo a olho nu permite detectar a presença de possíveis prejuízos na integridade do cateter venoso e o desprendimento de coágulos visíveis para a parte distal do PICC.

### Local do Estudo

O estudo foi desenvolvido em um laboratório de pós-graduação pertencente a uma universidade estadual do Paraná. O local foi utilizado, exclusivamente, para a coleta de dados. A captação dos cateteres ocorreu na unidade de terapia intensiva e de cuidados intermediários neonatal do hospital universitário.

### População e Amostra

A amostra foi composta por 90 cateteres utilizados por RN hospitalizados em uma unidade neonatal. Estes dispositivos receberam infusão de nutrição parenteral ou antibioticoterapia ou solução glicosada com concentração de infusão de glicose acima de 12 ou drogas vasoativas.

Após o término do tratamento, os dispositivos venosos foram retirados e avaliados quanto a sua integridade e a presença de dobras e torções para posteriormente serem lavados com 2 mL de água destilada em seringa de 10 mL para a garantia de sua permeabilidade. Além disso, foram colocados individualmente em sacos plásticos e armazenados em uma caixa opaca protegidos da luz e umidade. Para a identificação dos cateteres utilizados na pesquisa os mesmos foram etiquetados com as seguintes informações: datas de inserção e retirada, motivo de remoção, terapia infundida e comprimento em centímetros.

### Critérios de Seleção

Os critérios de inclusão foram cateteres de RN com calibre de 2 *French;* material de poliuretano; pertencer ao mesmo fabricante; pérvios; comprimento mínimo 11 centímetros, terem sido mantidos no RN por pelo menos sete e, no máximo, 30 dias; estar armazenados por, no máximo, dois anos e apresentarem em suas etiquetas de identificação todos os dados de caracterização. Foram necessários dois anos para que se atingisse o número de cateteres para compor o valor amostral. Foram excluídos os cateteres que não foram armazenados adequadamente e/ou que não estivessem pérvios e/ou não estivessem íntegros e/ou apresentassem dobras e/ou torções no momento de separação para a realização da pesquisa.

Foi necessário que os cateteres armazenados estivessem pérvios, pois o estudo objetivou simular, o mais próximo o possível, a técnica de desobstrução como acontece nas unidades neonatais. Na prática, a obstrução é identificada e, logo após, já são iniciadas as tentativas de desobstrução. Por ser um estudo experimental foram proporcionadas as mesmas condições ambientais para as soluções na tentativa de desobstrução dos PICC.

### Cálculo do Tamanho Amostral

Para a realização do cálculo amostral utilizou-se o prontuário eletrônico da unidade neonatal de referência do estudo para a coleta de informações a respeito da quantidade de PICC removidos por obstrução ou término de tratamento do último ano no período de 01 de maio de 2021 a 31 de maio de 2022, considerando que foi realizado, previamente, um estudo piloto no mês de junho. Foram retirados, no total, 121 PICC, o que resultou em um valor amostral de 82 cateteres para todos os três grupos, ressalta-se que foi considerado 5% de erro amostral e 95% de nível de confiança. Apesar do cálculo amostral referir 82 cateteres como margem de segurança, os pesquisadores optaram por estabelecer um número superior, ou seja, utilizou-se uma amostra de 90 cateteres.

### Coleta de Dados

Previamente à coleta de dados, foi realizado no mês de junho de 2022, um estudo piloto seguindo os mesmos critérios adotados para a coleta de dados, com 10% do valor total amostral, portanto, três cateteres em cada um dos três grupos, totalizando nove PICC.

No estudo piloto foi possível determinar o tempo necessário para que ocorresse a obstrução dos cateteres (8 horas). Em relação às tentativas de desobstrução foram empregadas as mesmas técnicas utilizadas na intervenção. Porém, percebeu-se no teste piloto que a seringa de 10 mL de *Luer-Lock* proporcionava maior pressão negativa e menos ocorrências de desconexões quando comparada com a seringa de 10 mL de *Luer-Slip*, portanto, o uso da seringa de 10 mL de *Luer-Lock* foi padronizado na intervenção.

Realizou-se o controle da temperatura e da umidade do laboratório por meio de um termômetro de ambiente, com intervalo de uma em uma hora, ao longo dos seis dias de coleta. A temperatura oscilou de 21,1^o^C a 25,1^o^C e a umidade variou de 37% a 56%.

Anteriormente ao início da intervenção, todos os dispositivos foram testados novamente com seringa de 10 mL e água destilada para a confirmação de sua permeabilidade e foram avaliadas também a sua integridade e a presença de dobras e torções – aqueles em que estas alterações estavam presentes foram descartados. Além disso, foi realizada uma nova checagem das informações constantes nas etiquetas dos cateteres e transferidas para uma planilha eletrônica. Em seguida, todos os cateteres foram cortados em 11 cm, por meio de lâmina de bisturi, para a padronização dos dispositivos, visto que na prática, utiliza-se a medida de 5 a 30 cm para a inserção. Na sequência, os dispositivos foram numerados e marcados com caneta permanente de 1 a 102 aleatoriamente. Todos os 102 PICC foram alocados em grupos de acordo com o tempo de permanência do RN, tempo de armazenamento e terapia infundida, o comprimento em centímetros do armazenamento não foi considerado, pois os cateteres foram todos cortados em 11 centímetros. Cabe ressaltar que foram incluídos 12 cateteres a mais do que a amostra total para garantir que não ocorresse prejuízo durante a coleta.

A coleta de dados foi realizada no mês de agosto de 2022 em seis dias diferentes de intervenção. A cada dia foram sorteados 17 cateteres aleatoriamente, assegurando que os grupos intervenção fossem os mais homogêneos possíveis de acordo com o tempo de permanência no RN, tempo de armazenamento e terapia infundida. Selecionou-se 17 cateteres para que se garantisse a obstrução de, pelo menos, 15 dispositivos por dia, distribuídos igualmente em três grupos. Em cada PICC foi aspirado sangue do mesmo doador voluntário para a sua obstrução.

Para a seleção do doador determinou-se que ele não fizesse nenhum tipo de tratamento de saúde e que não apresentasse alterações nos fatores de coagulação. O resultado dos exames coletados foram: tipagem sanguínea A+, tempo de tromboplastina parcial ativada de 34,3 segundos e relação P/N de 1,11; tempo de determinação de protrombina de 11,1 segundos; atividade de 116%; relação P/N de 0,94; plaquetas de 370 mil/µL; hemoglobina de 13,6 g/dL; hemácias de 4,42 milhões/µL; hematócrito de 40,1%; leucócitos totais de 7720 µ/L. Foi optado pela utilização de sangue de um doador adulto para que não houvesse danos decorrentes de punção para coleta de sangue de RN gravemente hospitalizado na unidade neonatal.

A cada dia foram coletados 3 mL de sangue do mesmo doador por meio de punção venosa com *Scalp* n^o^ 23 e seringa de 10 mL. O sangue foi colocado em um recipiente aberto, no qual a extremidade distal do cateter foi inserida para que ocorresse a aspiração de 0,05 mL (volume de *priming*) de sangue, através de seringa de 1 mL dos 17 cateteres. O tempo para a coleta e a aspiração do sangue foi de aproximadamente dois minutos.

Em seguida, por meio de uma pinça Kelly, realizou- se o clampeamento da extremidade distal dos cateteres e a extremidade proximal foi ocluída uma a uma com seringas de 1 mL para que não ocorresse a entrada de ar e/ou água destilada e/ou a saída do sangue do lúmen do PICC.

Os cateteres foram submersos em banho-maria com água destilada a 37^o^C (Quimis Aparelhos Científicos Ltda.) onde permaneceram por 8 horas na técnica *over night.* Optou-se por estabelecer a temperatura de 37^o^C buscando simular a temperatura considerada normotérmica do RN.

Para a garantia do controle térmico durante o período da noite, foram realizados testes de temperatura com o dispositivo antes da coleta de dados. A temperatura da água destilada oscilou de 36,0^o^C a 38,0^o^C. Para a garantia de que os cateteres não tocassem o fundo do dispositivo e pudesse ocorrer superaquecimento do PICC, foi colocada uma cuba rim no interior do aparelho para protegê-los.

Ao longo dos seis dias, os dispositivos venosos foram colocados em banho-maria entre os horários de 00h00-00h30 e permaneceram até os horários de 08h00-08h30.

Após as oito horas em banho-maria, foi cortado, com uso de bisturi, um centímetro da extremidade distal dos cateteres, que havia sido ocluída com a pinça Kelly, para garantir a integridade do dispositivo. Dessa forma, ao final, todos os PICC ficaram com 10 cm de comprimento. Em seguida, testou-se a oclusão por meio da tentativa de infusão de solução salina em seringa de 10 mL. A verificação da oclusão ocorreu por meio da resistência e não possibilidade de infusão da solução salina. A técnica de oclusão dos cateteres foi baseada em dois estudos realizados anteriormente^(16,17)^.

Foi elaborado um fluxograma com a descrição do método de obstrução dos cateteres venosos com sangue, conforme demonstrado na Figura 1.

**Figura 1:** Descrição do método de obstrução dos cateteres venosos centrais. Londrina, Paraná, 2022.

Autores da pesquisa, 2022.

Os cateteres que não obstruíram eram classificados como perdas e os 15 primeiros cateteres ocluídos foram inclusos na pesquisa. Caso houvesse mais de 15 PICC que estivessem obstruídos, eles foram descartados como perda, a fim de que a cada dia, cada grupo recebesse cinco cateteres de forma igualitária. A pesquisa teve quatro cateteres que não ocluíram e oito foram descartados para que ocorresse a homogeneidade entre os grupos.

Os 15 primeiros dispositivos obstruídos foram distribuídos aleatoriamente por meio de sorteio em três grupos. Cada grupo recebeu uma solução diferente para testar a sua capacidade de desobstrução, a distribuição dos números dos grupos ocorreu de forma aleatória por meio de sorteio.

O grupo 1 recebeu uma seringa de 10 mL preenchida somente com solução salina.

O grupo 2 recebeu vitamina C injetável na concentração de 25 mg/mL diluída em solução salina em seringa de 10 mL. Devido à ausência de estudos sobre uso da vitamina C injetável na desobstrução de cateteres em RN, a concentração da solução foi calculada com base na dose diária recomendada para bebês prematuros que é 15-25 mg/kg/dia^(18)^.

O grupo 3 recebeu heparina de baixo peso molecular na dose de 50 mg/mL diluído em solução salina em seringa de 10 mL. A dose da heparina de baixo peso molecular foi determinada por meio de um estudo experimental *in vitro* realizado previamente que determinou que a heparina de baixo peso molecular na concentração de 50 mg/mL foi eficaz na desobstrução do PICC de RN^(16)^.

As seringas com as soluções foram protegidas da luz por meio de papel laminado e receberam uma fita colorida, sendo determinada a cor por meio de sorteio para a diferenciação da solução que estava sendo testada. O soro fisiológico, grupo 1, recebeu fita na cor verde; a vitamina C, grupo 2, recebeu a fita de cor amarela; e a heparina, grupo 3, recebeu a fita de cor vermelha.

A técnica adotada para a desobstrução do PICC foi pressão negativa por meio da torneira de três vias, sendo que em uma via foi conectado o cateter obstruído, na extremidade proximal da torneira foi colocado uma seringa de *Luer-Lock* de 10 mL vazia e na extremidade distal da torneira de três vias foi colocada uma seringa de *Luer-Lock* de 10 mL com a solução em teste^(19)^.

Nesta técnica fecha-se a via da solução e realiza-se a aspiração do conteúdo do lúmen do cateter até a marca de 10 mL da seringa vazia; em seguida, fecha-se a via da seringa vazia e abre-se a via da seringa com solução, possibilitando que a solução preencha o cateter por meio da pressão negativa gerada no lúmen do PICC. Em nenhum momento é realizada a técnica de infusão, somente aspiração, dessa forma, objetiva-se a aspiração dos coágulos e da solução^(19)^.

A tentativa de desobstrução foi realizada com os cateteres submersos em banho-maria na água destilada a 37^o^C nos tempos de 1, 5, 10, 15, 30, 60, 120 e 240 minutos, sendo que em cada tempo foram realizadas três tentativas de desobstrução. Estes intervalos de tempo foram determinados para a melhor análise de progressão aritmética. A cada 240 minutos, se não ocorresse a recuperação da permeabilidade do PICC, era preparada uma nova solução, devido à instabilidade da heparina. Determinou-se o tempo máximo de desobstrução de 24 horas após a identificação da oclusão.

Foi considerada a recuperação da permeabilidade do cateter venoso quando foi observada a aspiração dos coágulos e da água destilada do banho-maria no qual estavam submersos. Para a confirmação da desobstrução infundiu-se 1 mL de solução salina em um dispositivo graduado no tempo de um minuto. Após o início da coleta de dados não foi necessária nenhuma alteração no protocolo do estudo.

### Randomização

A randomização simples foi realizada por um profissional externo à pesquisa, responsável por preparar três envelopes opacos, identificados com fitas nas cores verde, azul e amarela, que determinaram a qual grupo cada solução pertencia. Esse mesmo profissional também identificou as seringas com as respectivas cores e soluções correspondentes. A ordem de seleção dos PICC para a tentativa de desobstrução foi determinada por meio de randomização simples, conforme demonstrado no fluxograma da Figura 2.

**Figura 2:** Descrição da aleatorização dos cateteres venosos centrais obstruídos para o início das tentativas de desobstrução. Londrina, Paraná, 2022.

Autores da pesquisa, 2022.

### Cegamento

A coleta de dados contou com a participação de dois pesquisadores, sendo que o pesquisador 1 realizou a tentativa de desobstrução de todos os cateteres ao longo dos seis dias e o pesquisador 2 foi o responsável em randomizar as soluções a serem testadas em cada cateter. O cegamento ocorreu por parte do pesquisador 1 que era quem realizava a técnica de pressão negativa para desobstruir o PICC; e pelo responsável em realizar a análise estatística dos dados.

Em uma planilha eletrônica anotou-se o número do cateter, o número da solução (1, 2, 3), número de tentativas e tempo de desobstrução. Foi realizada a dupla digitação e dupla conferência dos dados.

### Análise e Tratamento dos Dados

Os testes foram realizados no programa *Statistical Package for the Social Sciences* (SPSS (25.0)). A análise descritiva foi apresentada por meio de média e desvio padrão (DP). As variáveis do número de tentativas e do tempo para desobstrução foram avaliadas por meio da análise de normalidade dos dados através do teste de *Shapiro-Wilk*. As variáveis apresentaram distribuição anormal, portanto, a diferença entre os grupos de intervenção foi avaliada por meio do Teste de *Kruskal-Wallis.* Foi adotado nível de significância de 5,0% e p ≤ 0,05.

### Aspectos Éticos

Este estudo integra-se ao projeto de pesquisa intitulado “Barreiras e potencialidades no cuidado com o cateter central de inserção periférica em neonatologia: buscando inovações”. A pesquisa teve aprovação do Comitê de Ética em Pesquisa, sob n° CEP/UEL: 4.324.095. CAAE: 38696720.9.0000.5231 e foi realizada de acordo com os preceitos éticos necessários.

Foi dispensada a necessidade de aprovação dos pais e/ou responsáveis dos RN através de assinatura do termo de consentimento livre e esclarecido (TCLE) para a utilização do PICC que estava em seu filho, pois o dispositivo venoso seria descartado, já que a sua remoção ocorreu após o término do tratamento. Além disso, por ter sido realizado *flush* com 2 mL de água destilada, os cateteres não continham material biológico do paciente.

## RESULTADOS

Foi elaborada a Tabela 1 que contém a caracterização dos 90 PICC que compuseram a amostra. Observa-se que o comprimento de armazenamento do cateter mais frequente foi de 16 a 20 cm, a maior parte dos cateteres foram armazenados pelo período de 0 a 6 meses, de 7 a 12 dias foi o período de permanência no RN com maior prevalência, a maioria dos dispositivos receberam a infusão de antibioticoterapia e todos os cateteres foram removidos devido ao término do tratamento.

**Tabela 1:** Caracterização dos PICC que compuseram a amostra – Londrina, PR, Brasil, 2022.

Características	Cateteres que compuseram a amostra (n = 90)
Comprimento do cateter no armazenamento	24,45% (11 a 15 cm)
	31,12% (16 a 20 cm)
	27,78% (21 a 25 cm)
	16,65% (26 a 30 cm)
Período de armazenamento do cateter	31,12% (0 a 6 meses)
	18,88% (7 a 12 meses)
	27,77% (13 a 18 meses)
	22,23% (19 a 24 meses)
Tempo de permanência no RN	62,06% (7 a 12 dias)
	24,14% (13 a 18 dias)
	6,90% (19 a 24 dias)
	6,90% (25 a 30 dias)
Tipo de terapia infundida	60,65% (antibioticoterapia)
	31,14% (nutrição parenteral)
	4,92% (droga vasoativa)
	3,29% (solução glicosada com concentração de infusão de glicose acima de 12)
Motivo de retirada	100% (término do tratamento)

Fonte: Dados da pesquisa.

Observa-se na Tabela 2 que as médias e desvio-padrão em relação às tentativas de desobstrução foram de 8,47 (5,23) do soro fisiológico (substância 1), 9,43 (5,61) da vitamina C (substância 2), 8,20 (5,59) da heparina (substância 3) e com p-valor de 0,639 pelo *Kruskal-Wallis.* Já em relação ao tempo de desobstrução em minutos, as médias e desvio-padrão foram de 30,80 (40,63) do soro fisiológico (substância 1), 35,43 (45,68) da vitamina C (substância 2), 29,17 (41,17) da heparina (substância 3) e com p-valor de 0,758 pelo *Kruskal-Wallis.*

**Tabela 2:** Avaliação da diferença entre os grupos de intervenção associado ao número de tentativas e o tempo em minutos – Londrina, PR, Brasil, 2022.

Variáveis, X* (DP)**	Total	Soluções			p-valor^†^
		1^††^	2^†††^	3^††††^	
Tentativas de desobstrução	8,70 (5,45)	8,47 (5,23)	9,43 (5,61)	8,20 (5,59)	0,639
Tempo (minutos)	31,80 (42,16)	30,80 (40,63)	35,43 (45,68)	29,17 (41,17)	0,758

X*: média.

DP**: Desvio-padrão.

^†^Teste de Kruskal-Wallis.

1^††^: soro fisiológico.

2^†††^: vitamina C.

3^††††^: heparina.

Fonte: Dados da pesquisa.

A Tabela 3 apresenta a descrição das soluções na desobstrução dos PICC em relação aos intervalos de tempo e às tentativas. Observa-se que a quantidade de cateteres desobstruídos por cada uma das três soluções foi similar ao longo de cada período de tempo (1, 5, 10 15, 30, 60 e 120 minutos). O tempo máximo de desobstrução dos cateteres pelas três soluções foi de 120 minutos, porém não foram necessárias as segunda e terceira tentativas de desobstrução no tempo de 120 minutos para a solução de soro fisiológico, diferentemente das soluções de vitamina C e de heparina.

**Tabela 3:** Desobstrução dos cateteres ocluídos em laboratório de acordo com as três soluções em relação ao tempo e à tentativa – Londrina, PR, Brasil, 2022.

Tempo	Tentativa	Solução 1 (soro fisiológico)	Solução 2 (vitamina C)	Solução 3 (heparina)
		Quantidade de cateteres desobstruídos (n)		
1 minuto	1ª	1	0	2
	2ª	2	2	2
	3ª	6	6	6
Subtotal		9/30 (30%)	8/30 (26,66%)	10/30 (33,33%)
5 minutos	1ª	1	0	1
	2ª	2	2	3
	3ª	1	0	1
Subtotal		13/30 (43,33%)	10/30 (33,33%)	15/30 (50%)
10 minutos	1ª	2	2	0
	2ª	1	2	1
	3ª	1	3	2
Subtotal		17/30 (56,66%)	17/30 (56,66%)	18/30 (60%)
15 minutos	1ª	0	1	0
	2ª	0	0	1
	3ª	1	0	1
Subtotal		18/30 (60%)	18/30 (60%)	20/30 (66,66%)
30 minutos	1ª	0	1	0
	2ª	1	0	1
	3ª	3	3	1
Subtotal		22/30 (73,33%)	22/30 (73,33%)	22/30 (73,33%)
60 minutos	1ª	1	0	1
	2ª	1	0	2
	3ª	2	2	1
Subtotal		26/30 (86,66%)	24/30 (80%)	26/30 (86,66%)
120 minutos	1ª	4	0	1
	2ª	–	2	1
	3ª	–	4	2
Total		30/30 (100%)	30/30 (100%)	0/30 (100%)

Fonte: Dados da pesquisa.

Durante as tentativas de desobstrução não foi observado a olho nu a presença de possíveis prejuízos na integridade do PICC e nem o desprendimento de coágulos visíveis para a sua parte distal, mas foi possível visualizar a aspiração de coágulo para dentro da seringa na qual foi realizada a pressão negativa.

## DISCUSSÃO

Ao avaliar a eficácia das soluções de soro fisiológico, heparina e vitamina C na desobstrução de cateter venoso central neonatal ocluído por coágulos, tem-se que as três substâncias apresentaram eficácia para desobstrução dos cateteres por coágulos.

A obstrução por coágulos dentro do lúmen do PICC pode ocorrer de forma parcial ou total ocasionado pela presença de fibrina ou trombos sanguíneos, provocando a dificuldade ou impossibilidade em administrar as medicações devido à ausência de permeabilidade. Geralmente, está associada ao fluxo retrógrado dentro do cateter ou à falha na realização do *flushing* ^(20)^. O trombo consiste em uma das principais causas de obstrução de dispositivos venosos centrais. A formação do trombo acontece pela chamada tríade de *Virchow*: estase sanguínea, lesão do endotélio e hipercoagulabilidade^(21)^.

Esta pesquisa demonstrou que o soro fisiológico foi tão eficaz quanto a heparina na concentração de 50 mg/mL e a vitamina C na concentração de 25 mg/mL para a desobstrução de PICC coagulados por sangue *in vitro* por meio do uso da técnica de pressão negativa.

O soro fisiológico é composto por uma solução aquosa com concentração a 0,9% de cloreto de sódio e que pode ser utilizado por via intravenosa, intraocular, inalatória, dentre outras. É um composto com alta estabilidade no aspecto microbiológico como também físico-químico^(22)^. Dentre os cristaloides, o soro fisiológico 0,9% é o mais utilizado, apesar de possuir concentrações de cloro e sódio elevadas em relação ao plasma. Possui osmolaridade maior, porém próxima a do sangue. Após a infusão desta solução, o cloro e o sódio ficam no líquido extracelular, o que permite considerar o soro fisiológico como uma solução isotônica^(23)^. São desconhecidos os efeitos anticoagulantes do soro fisiológico.

A vitamina C consiste em um essencial micronutriente que faz parte de vários processos biológicos e bioquímicos. Há duas apresentações da vitamina C no sangue: dehidroascorbato (forma oxidada) e ácido ascórbico. Essa vitamina age através do bloqueio da adesão das plaquetas no endotélio vascular e por meio da agregação plaquetária que é mediada pela P-selectina e induzida pela trombina^(24)^.

Corroborando com os achados deste estudo, pesquisas randomizadas comparando a vitamina C e o soro fisiológico têm demonstrado que não há diferença significativa em relação a essas duas soluções na prevenção de oclusão de cateteres venosos centrais^(12,25)^.

Diante de pequenos coágulos, a infusão do ácido ascórbico desobstrui o vaso sanguíneo e remove a adesão das plaquetas no endotélio. Além disso, a vitamina C atua inibindo a liberação que é provocada pela trombina que depende do pH o ativador do plasminogênio-inibidor-1 de plaquetas. Ela também reduz as concentrações do fator de *Willebrand* e o ativador de plasminogênio tecidual demonstrando um importante papel na coagulação e inflamação^(24)^. Faltam evidências científicas que abordem os efeitos adversos da vitamina C na população neonatal.

A heparina é formada por cadeias de polissacarídeos de vários pesos moleculares e possui alta afinidade com o inibidor fisiológico de coagulação, antitrombina, fator X ativado (Xa) e trombina (IIa). Neste estudo utilizou-se a heparina de baixo peso molecular, sua função anticoagulante é realizada por meio da antitrombina III que age através da inibição dos fatores de coagulação IXa, XIa, em menor escala, e XIIa e IIa e Xa em maior intensidade^(26)^. A enoxaparina é a heparina de baixo peso molecular mais utilizada, pois precisa de uma dose menor para Xa que atinja valores do adulto e tem uma depuração menor em neonatos. Dentre os efeitos adversos, destaca-se o sangramento^(27)^.

Corroborando com os achados deste estudo, estudos randomizados analisaram que a heparina e o soro fisiológico não apresentaram diferença estatística significativa em relação à eficácia na manutenção da permeabilidade de dispositivos venosos centrais, portanto, foi recomendado o uso de solução salina^(28,29)^.

Já em relação à desobstrução de dispositivos venosos obstruídos por coágulos, existe a recomendação da utilização da heparina, principalmente no cenário nacional^(16)^ e da alteplase, especialmente no cenário internacional^(14)^. Até o ano de 1999, nos Estados Unidos recomendavam a utilização da uroquinase, porém, a *Food and Drug Administration* (FDA) proibiu a utilização deste fármaco devido aos riscos na sua segurança e eficácia, pois foi associada a eventos de hemorragia grave^(14,30)^.

É importante considerar que em RN enfermos há descontrole dos níveis desses fatores de coagulação que podem estar relacionados a várias causas diferentes, como estado pró- coagulante, dano endotelial e redução do fluxo, favorecendo a formação de trombos ou a ocorrência de sangramentos^(27)^.

Este estudo utilizou a técnica da pressão negativa, porém, na literatura encontram-se duas técnicas para a desobstrução de cateteres centrais: a infusão de soluções e a técnica da pressão negativa^(31)^.

Quando ocorre oclusão parcial do PICC, detectada por aumento da resistência na administração de medicamentos, é recomendado a técnica de infusão da medicação com a solução para recuperar a permeabilidade. No caso de oclusão total do PICC, deve-se utilizar a técnica de pressão negativa para minimizar o risco de danos^(31)^.

Como a obstrução pode acontecer em qualquer segmento interno do PICC, é possível que o cateter possua solução entre a obstrução e a extremidade proximal, dessa forma a técnica da pressão negativa tem como objetivo retirar esta solução e provocar uma pressão negativa intraluminal no dispositivo venoso. Quando ocorre a liberação da pressão negativa, provoca-se a aspiração da substância presente na outra seringa que está conectada ao cateter por meio da torneira de três vias^(32)^.

Esta técnica reduz os danos que a sobrepressurização pode provocar no PICC e possibilita que a solução responsável em desobstruir o cateter atinja mais rapidamente o trombo causador da oclusão, pois não é necessário forçar o êmbolo da seringa para que a solução adentre na região intraluminal do dispositivo venoso^(32)^.

A manipulação do cateter deve ser realizada com seringa de, no mínimo, 10 mL para que não provoque pressão superior a 25 psi dentro do cateter, evitando ruptura do cateter e a ocorrência de danos na rede venosa^(33)^.

Sabe-se que a obstrução de um cateter venoso traz uma série de consequências e impactos negativos para o RN e sua família, dentre elas, a cessação na terapia medicamentosa, aumento de custos hospitalares, estresse, aumento de procedimentos dolorosos e inserções repetidas para um novo dispositivo venoso^(34)^. Ressalta-se que o PICC em RN possui maiores chances de oclusão em decorrência de seu pequeno calibre^(12)^.

Ensaios clínicos randomizados demonstram ausência de evidências científicas que consigam garantir a recomendação de utilizar a heparina^(35)^ o soro fisiológico e o ácido ascórbico^(12)^ para a manutenção da permeabilidade do cateter venoso central. Corroborando com os achados desta pesquisa de que não houve diferença estatística significativa entre as soluções para a desobstrução dos PICC.

Diversas estratégias foram desenvolvidas objetivando a prevenção de obstrução de cateteres venosos, como a criação de uma válvula antirrefluxo que é colocada na extremidade distal do PICC a fim de evitar o refluxo de sangue para dentro do lúmen do cateter, porém, estudos científicos não demonstraram redução da taxa de ocorrência de oclusão e, em contrapartida, é uma estratégia que eleva o custo com o dispositivo venoso^(36)^.

Quanto aos cuidados com cateteres venosos, destaca-se a equipe de enfermagem, principalmente o enfermeiro, que é o profissional responsável em definir e realizar o curativo melhor apropriado para o paciente, além de realizar medidas de prevenção de infecção e obstrução. Cabe ao enfermeiro garantir os cuidados na manipulação, avaliar diariamente os dispositivos venosos e realizar capacitações à equipe de saúde responsável pelos cuidados com o RN^(37)^.

Dentre os cuidados a serem realizados para a prevenção da oclusão tem-se a realização da instilação de soro fisiológico com pressão positiva para evitar que ocorra retorno de sangue para dentro do lúmen do cateter^(19)^, que deve ser feita de seis em seis horas ou antes e após a infusão de medicações. Para isto é necessário fazer a infusão de soro fisiológico por meio da técnica pulsátil a fim de que cause turbilhonamento no interior do cateter para que haja a remoção de depósito de fibrina^(12)^.

Considera-se como limitação que não foram realizadas análises microscópicas da parte interna dos cateteres com o objetivo de averiguar a desobstrução parcial ou total, deste modo, a verificação da desobstrução foi feita apenas por olhar clínico, ou seja, foi considerado desobstruído o cateter que ao ser instilado solução salina na região proximal, foi observado o fluido em sua parte distal sem que ocorresse resistência para sua infusão. Também pode ser considerado como um fator limitante, os dois anos de captação e armazenamento que foram necessários para que se pudessem atingir a quantidade suficiente de PICC para atingir o valor amostral, pois neste tempo, na unidade neonatal a qual está vinculada esta pesquisa, havia cateteres que foram retirados por obstrução e ruptura e que, portanto, não poderiam ser incluídos nesta pesquisa.

Estudos *in vitro* são essenciais para investigar mecanismos biológicos e testar inicialmente a eficácia e segurança de procedimentos em ambiente controlado. Contudo, a validade desses estudos para aplicação direta em humanos, especialmente em neonatologia, é limitada, pois não reproduzem integralmente a complexidade do organismo vivo, incluindo respostas imunológicas, metabolismo e interações celulares sistêmicas. Por isso, os achados *in vitro* devem ser interpretados como um passo preliminar que necessita confirmação por meio de estudos *in vivo* e clínicos, garantindo segurança e eficácia reais para os pacientes neonatais^(38-40)^.

Além disso, a extrapolação direta dos resultados *in vitro* para a prática clínica requer cautela, pois fatores como o ambiente fisiológico, variabilidade individual e condições clínicas específicas dos recém-nascidos podem influenciar o comportamento do dispositivo ou da substância testada. Portanto, para que intervenções baseadas em dados *in vitro* sejam adotadas na assistência de enfermagem neonatal, é imprescindível a realização de pesquisas clínicas rigorosas que validem esses achados e promovam a prática baseada em evidências, assegurando a qualidade e a segurança do cuidado^(38,39)^.

Espera-se que esta pesquisa sirva de base para futuros estudos que explorem alternativas mais seguras e eficazes para a desobstrução de PICC em RN, especialmente no que diz respeito à comparação entre diferentes soluções e técnicas utilizadas na prática clínica. Entre as principais lacunas a serem abordadas em pesquisas futuras destacam-se a avaliação dos efeitos adversos a longo prazo, a padronização de protocolos de manejo e a análise do custo-benefício das intervenções. Do ponto de vista prático, os resultados deste estudo podem subsidiar a assistência de enfermagem, promovendo condutas mais seguras e baseadas em evidências, o que contribui para a redução de complicações e para a melhoria dos desfechos clínicos dos neonatos. Ressalta-se, ainda, a importância da adoção de práticas em saúde fundamentadas em evidências científicas, garantindo uma assistência qualificada e centrada nas necessidades dos pacientes.

## CONCLUSÃO

As três substâncias utilizadas apresentaram eficácia para desobstrução dos cateteres por coágulos, visto que todos os cateteres tiveram suas permeabilidades reestabelecidas com tempo máximo de 120 minutos, por meio da técnica de pressão negativa. Este tempo de até 120 minutos é viável na prática clínica, visto que o protocolo de desobstrução de cateteres venosos centrais da unidade neonatal, vinculada a esse estudo, considera o tempo máximo de 24 horas para a recuperação da permeabilidade destes dispositivos.

Desta forma, a hipótese desse estudo foi refutada uma vez que não houve significância estatística entre as soluções utilizadas para desobstrução dos PICC em relação ao tempo e ao número de tentativas.

Recomenda-se a utilização do soro fisiológico, pois possui estabilidade físico-química e microbiana, trazendo menores efeitos colaterais em relação ao seu uso em RN, principalmente, quando comparado a outras soluções.

## Data Availability

Todo o conjunto de dados que dá suporte aos resultados deste estudo foi publicado no próprio artigo.

# Translation: Evaluación de la eficacia de soluciones para desobstruir catéteres venosos centrales neonatales: estudio aleatorizado*

**DOI:** 10.1590/1980-220X-REEUSP-2025-0122es

**Authors:** Carolina Mathiolli, Juliane Pagliari Araujo, Keli Regiane Tomeleri da Fonseca Pinto, Rosangela Aparecida Pimenta, Danielle Venturini, Sonia Silva Marcon, Adriana Valongo Zani

**Affiliations:** 1 Universidade Estadual de Londrina, Departamento de Enfermagem, Londrina, PR, Brasil.; 2 Instituto Federal do Paraná, Departamento de Enfermagem, Londrina, PR, Brasil.; 3 Universidade Estadual de Londrina, Departamento de Patologia Aplicada, Análise Clínicas e Toxicológicas, Londrina, PR, Brasil.; 4 Universidade Estadual de Maringá, Departamento de Enfermagem, Maringá, PR, Brasil.

**Keywords:** Obstrucción del catéter, Recién Nacido, Heparina, Ácido ascórbico, Solución Salina

## Abstract

**Objetivo:**

Evaluar la eficacia de las soluciones de suero fisiológico, heparina y vitamina C en la desobstrucción de catéteres venosos centrales neonatales ocluidos por coágulos.

**Método:**

Estudio experimental doble ciego aleatorizado *in vitro* realizado en agosto de 2022 con 90 catéteres venosos centrales neonatales ocluidos con sangre que permanecieron en baño María durante 8 horas. Las soluciones probadas fueron: solución salina, heparina (50 mg/mL) y vitamina C (25 mg/mL), mediante la técnica de presión negativa. Las variables del número de intentos y el tiempo de desobstrucción se evaluaron en relación con la media y la desviación estándar. Para las variables con distribución anormal se utilizó la prueba de Kruskal-Wallis.

**Resultados:**

No hubo significación estadística en relación con el tiempo y el número de intentos de desobstrucción de las tres soluciones (p > 0,05) y el tiempo máximo de desobstrucción de los catéteres fue de 120 minutos.

**Conclusión:**

Las tres sustancias presentaron resultados similares. Por lo tanto, se recomienda la solución salina por considerarse segura y tener menos efectos secundarios en relación con su uso en recién nacidos.

## INTRODUCCIÓN

El catéter central de inserción periférica (*peripherally inserted central catheter*, PICC) es un dispositivo largo y flexible que se inserta a través de una punción venosa periférica y llega hasta la porción distal de la vena cava inferior o superior, según el lugar de inserción^(1,2)^.

De acuerdo con la Resolución 258 de 2001, el enfermero cuenta con el respaldo del Consejo Federal de Enfermería para la indicación, inserción, mantenimiento y retirada del PICC, siempre que posea la habilitación técnica y teórica para ello, ya que se trata de un procedimiento de alta complejidad. Ante esto, el enfermero es uno de los principales responsables del control de infecciones y la prevención de obstrucciones de este dispositivo venoso de larga permanencia, especialmente en la población neonatal^(3)^.

El PICC está especialmente indicado para recién nacidos (RN), ya que el catéter está fabricado con materiales de baja trombogénesis (poliuretano y silicona), lo que proporciona un tiempo de terapia intravenosa prolongado y seguro, además de reducir el número de punciones y, en consecuencia, los procedimientos dolorosos^(4,5)^. Para la población neonatal, las principales indicaciones para elegir este dispositivo están relacionadas con la necesidad de terapia intravenosa durante seis días o más, la infusión de soluciones hiperosmolares, sedantes, fármacos vasoactivos, nutrición parenteral total o parcial, quimioterapéuticos y medicamentos vesicantes y/o irritantes^(6)^.

A pesar de sus beneficios, el uso de este dispositivo puede acarrear complicaciones que pueden perjudicar el estado de salud del recién nacido. Las tasas de ocurrencia de complicaciones varían entre el 32.8 y el 48.8%, siendo la obstrucción la más frecuente (5.55 a 21.40%) y, con porcentajes menores, la infección (1,85 a 14,30%), la rotura (1,1 a 13,04%), la infiltración (4,34 a 12,03%) y la extracción accidental (2,4 a 11,11%)^(7)^.

Entre las complicaciones, destaca la obstrucción del catéter. Esta investigación se centra en la obstrucción por coágulos, que se produce debido a su presencia en el interior de la luz o en el extremo distal, pudiendo ocluir de forma parcial o total. Se evidencia por la dificultad para infundir medicamentos o aspirar sangre a través del dispositivo. La incidencia de obstrucción en el recién nacido varía del 9,7% al 30,9%^(8,9)^.

La prevención de la oclusión se considera la mejor estrategia de cuidado para el catéter venoso central, lo que puede garantizarse mediante el lavado con solución salina antes y después de las infusiones de sustancias. Sin embargo, algunas publicaciones indican la infusión continua de 0,5 U/kg de heparina en solución salina^(10)^. Por el contrario, una revisión sistemática presentó en sus resultados la ausencia de evidencia científica para afirmar que existe una diferencia entre el lavado con heparina y el lavado con solución salina para el mantenimiento del acceso venoso central en recién nacidos y niños^(11)^. Corroborando esta investigación, un ensayo clínico aleatorio demostró que no hubo diferencias estadísticas entre el lavado con vitamina C y el lavado con suero fisiológico en la prevención de la obstrucción del catéter venoso central en niños^(12)^. Además, los autores afirman que faltan estudios que establezcan la seguridad, eficacia y eficiencia de las soluciones^(10)^.

Aunque faltan pruebas científicas para la prevención de la obstrucción y la restauración de la permeabilidad del PICC, se sabe que en la práctica diaria se utilizan varios tipos de soluciones para desobstruir catéteres venosos centrales con el objetivo de recuperar estos dispositivos^(13,14)^ debido a la imposibilidad de un nuevo acceso venoso central como consecuencia de la inestabilidad hemodinámica y/o la dificultad de la red venosa.

Ante esto, se elaboró la pregunta de investigación de acuerdo con la estrategia mnemónica PICO (Población (P), Intervención (I), Comparación (C) y Resultado (O)), siendo P: catéter venoso central de inserción periférica de recién nacido ocluido por coágulos, I: infusión de suero fisiológico, C: infusión de heparina e infusión de vitamina C y O: desobstrucción del catéter. «¿Cuál es la eficacia del suero fisiológico en comparación con la heparina y la vitamina C en la desobstrucción de catéteres venosos centrales de inserción periférica en recién nacidos ocluidos por coágulos?».

La hipótesis de este estudio es: ¿Existe alguna diferencia en la eficacia entre el suero fisiológico, la heparina y la vitamina C en la desobstrucción de catéteres venosos centrales de inserción periférica en recién nacidos ocluidos por coágulos? Por lo tanto, el objetivo fue evaluar la eficacia de las soluciones de solución salina, heparina y vitamina C en la desobstrucción de catéteres venosos centrales de inserción periférica en recién nacidos ocluidos por coágulos.

## MÉTODO

### Tipo de Estudio

Se trata de un estudio experimental doble ciego aleatorizado*in vitro*. Los estudios experimentales*in vitro*consisten en la realización de un experimento desarrollado en un entorno de laboratorio controlado, con el fin de obtener datos sobre la seguridad y la eficacia de un procedimiento en prueba, ante la imposibilidad de realizarlo en individuos vivos^(15)^.

Teniendo en cuenta que en la unidad neonatal se encuentra una población de pacientes graves, las intervenciones realizadas no deben perjudicar aún más su pronóstico. Por lo tanto, para establecer las dosis mínimas necesarias de las sustancias empleadas en la desobstrucción de los accesos venosos, sería inadecuado, en un principio, que la experimentación se realizara in vivo. Además, la evaluación del dispositivo en un entorno extracorpóreo a simple vista permite detectar la presencia de posibles daños en la integridad del catéter venoso y el desprendimiento de coágulos visibles en la parte distal del PICC.

### Lugar del Estudio

El estudio se llevó a cabo en un laboratorio de posgrado perteneciente a una universidad estatal de Paraná. El lugar se utilizó exclusivamente para la recopilación de datos. La obtención de los catéteres se realizó en la unidad de terapia intensiva y cuidados intermedios neonatales del hospital universitario.

### Población y Muestra

La muestra estuvo compuesta por 90 catéteres utilizados por recién nacidos hospitalizados en una unidad neonatal. Estos dispositivos recibieron infusión de nutrición parenteral o antibioterapia o solución glucosada con una concentración de infusión de glucosa superior a 12 o fármacos vasoactivos.

Una vez finalizado el tratamiento, se retiraron los dispositivos venosos y se evaluó su integridad y la presencia de pliegues y torceduras, para posteriormente lavarlos con 2 ml de agua destilada en una jeringa de 10 ml para garantizar su permeabilidad. Además, se colocaron individualmente en bolsas de plástico y se almacenaron en una caja opaca protegida de la luz y la humedad. Para identificar los catéteres utilizados en la investigación, se etiquetaron con la siguiente información: fechas de inserción y retirada, motivo de la retirada, terapia infundida y longitud en centímetros.

### Criterios de Selección

Los criterios de inclusión fueron catéteres para recién nacidos con un calibre de 2*French;*material de poliuretano; pertenecer al mismo fabricante; permeables; longitud mínima de 11 centímetros; haber permanecido en el recién nacido durante al menos siete y, como máximo, 30 días; haber sido almacenados durante un máximo de dos años y presentar en sus etiquetas de identificación todos los datos de caracterización. Se necesitaron dos años para alcanzar el número de catéteres necesarios para componer el valor muestral. Se excluyeron los catéteres que no se almacenaron adecuadamente y/o que no eran permeables y/o no estaban intactos y/o presentaban pliegues y/o torceduras en el momento de la separación para la realización de la investigación.

Era necesario que los catéteres almacenados fueran permeables, ya que el estudio tenía como objetivo simular, en la medida de lo posible, la técnica de desobstrucción tal y como se realiza en las unidades neonatales. En la práctica, se identifica la obstrucción y, a continuación, se inician los intentos de desobstrucción. Al tratarse de un estudio experimental, se proporcionaron las mismas condiciones ambientales para las soluciones en el intento de desobstrucción de los PICC.

### Cálculo del Tamaño de la Muestra

Para realizar el cálculo de la muestra, se utilizó el historial electrónico de la unidad neonatal de referencia del estudio para recopilar información sobre la cantidad de PICC retirados por obstrucción o finalización del tratamiento durante el último año, en el periodo comprendido entre el 1 de mayo de 2021 y el 31 de mayo de 2022, teniendo en cuenta que previamente se había realizado un estudio piloto en el mes de junio. Se retiraron un total de 121 PICC, lo que dio como resultado un valor muestral de 82 catéteres para los tres grupos, cabe destacar que se consideró un error muestral del 5% y un nivel de confianza del 95%. Aunque el cálculo de la muestra indicaba 82 catéteres como margen de seguridad, los investigadores optaron por establecer un número superior, es decir, se utilizó una muestra de 90 catéteres.

### Recolcción de Datos

Antes de la recolección de datos, en junio de 2022 se realizó un estudio piloto siguiendo los mismos criterios adoptados para la recopilación de datos, con el 10% del valor total de la muestra, es decir, tres catéteres en cada uno de los tres grupos, lo que suma un total de nueve PICC.

En el estudio piloto se pudo determinar el tiempo necesario para que se produjera la obstrucción de los catéteres (8 horas). En cuanto a los intentos de desobstrucción, se emplearon las mismas técnicas utilizadas en la intervención. Sin embargo, en la prueba piloto se observó que la jeringa de 10 ml de Luer-Lock proporcionaba una mayor presión negativa y menos desconexiones en comparación con la jeringa de 10 ml de Luer-Slip, por lo que se estandarizó el uso de la jeringa de 10 ml de Luer-Lock en la intervención.

Se controló la temperatura y la humedad del laboratorio mediante un termómetro ambiental, con intervalos de una hora, durante los seis días de recolección. La temperatura osciló entre 21,1 °C y 25,1 °C y la humedad varió entre el 37% y el 56%.

Antes del inicio de la intervención, todos los dispositivos se volvieron a probar con una jeringa de 10 ml y agua destilada para confirmar su permeabilidad, y también se evaluó su integridad y la presencia de pliegues y torceduras; aquellos en los que se observaron estas alteraciones fueron descartados. Además, se realizó una nueva verificación de la información que figuraba en las etiquetas de los catéteres y se transfirió a una hoja de cálculo electrónica. A continuación, todos los catéteres se cortaron a 11 cm con una cuchilla de bisturí para estandarizar los dispositivos, ya que en la práctica se utiliza una medida de 5 a 30 cm para la inserción. A continuación, los dispositivos se numeraron y marcaron con rotulador permanente del 1 al 102 de forma aleatoria. Los 102 PICC se distribuyeron en grupos según el tiempo de permanencia del recién nacido, el tiempo de almacenamiento y la terapia infundida; no se tuvo en cuenta la longitud en centímetros del almacenamiento, ya que todos los catéteres se cortaron a 11 centímetros. Cabe destacar que se incluyeron 12 catéteres más que la muestra total para garantizar que no se produjeran daños durante la recolección.

La recolección de datos se realizó en agosto de 2022 en seis días diferentes de intervención. Cada día se seleccionaron al azar 17 catéteres, asegurando que los grupos de intervención fueran lo más homogéneos posible según el tiempo de permanencia en el recién nacido, el tiempo de almacenamiento y la terapia infundida. Se seleccionaron 17 catéteres para garantizar la obstrucción de al menos 15 dispositivos por día, distribuidos equitativamente en tres grupos. En cada PICC se aspiró sangre del mismo donante voluntario para su obstrucción.

Para la selección del donante, se determinó que no recibiera ningún tipo de tratamiento médico y que no presentara alteraciones en los factores de coagulación. Los resultados de los exámenes recogidos fueron: grupo sanguíneo A+, tiempo de tromboplastina parcial activada de 34,3 segundos y relación P/N de 1,11; tiempo de protrombina de 11,1 segundos; actividad del 116%; relación P/N de 0,94; plaquetas de 370 mil/µL; hemoglobina de 13,6 g/dL; glóbulos rojos de 4,42 millones/µL; hematocrito de 40,1%; leucocitos totales de 7720 µ/L. Se optó por utilizar sangre de un donante adulto para que no hubiera daños derivados de la punción para la extracción de sangre de un recién nacido gravemente hospitalizado en la unidad neonatal.

Cada día se extrajeron 3 ml de sangre del mismo donante mediante punción venosa con una aguja Scalp n.º 23 y una jeringa de 10 ml. La sangre se colocó en un recipiente abierto, en el que se insertó el extremo distal del catéter para aspirar 0,05 ml (volumen de cebado) de sangre, mediante una jeringa de 1 ml de los 17 catéteres. El tiempo para la extracción y aspiración de la sangre fue de aproximadamente dos minutos.

A continuación, mediante una pinza Kelly, se realizó el pinzamiento del extremo distal de los catéteres y el extremo proximal se ocluyó uno a uno con jeringas de 1 ml para que no entrara aire y/o agua destilada y/o saliera sangre del lumen del PICC.

Los catéteres se sumergieron en un baño María con agua destilada a 37 °C (Quimis Aparelhos Científicos Ltda.), donde permanecieron durante 8 horas en la técnica*over night.*Se optó por establecer la temperatura de 37 °C con el fin de simular la temperatura considerada normotérmica del recién nacido.

Para garantizar el control térmico durante el período nocturno, se realizaron pruebas de temperatura con el dispositivo antes de la recopilación de datos. La temperatura del agua destilada osciló entre 36,0 °C y 38,0 °C. Para garantizar que los catéteres no tocaran el fondo del dispositivo y se pudiera producir un sobrecalentamiento del PICC, se colocó una cubeta en el interior del aparato para protegerlos.

A lo largo de los seis días, los dispositivos venosos se colocaron en un baño María entre las 00:00 y las 00:30 horas y permanecieron allí hasta las 08:00–08:30 horas.

Después de ocho horas en baño María, se cortó con un bisturí un centímetro del extremo distal de los catéteres, que había sido ocluido con la pinza Kelly, para garantizar la integridad del dispositivo. De esta manera, al final, todos los PICC quedaron con 10 cm de longitud. A continuación, se probó la oclusión mediante un intento de infusión de solución salina en una jeringa de 10 ml. La verificación de la oclusión se realizó mediante la resistencia y la imposibilidad de infundir la solución salina. La técnica de oclusión de los catéteres se basó en dos estudios realizados anteriormente^(16,17)^.

Se elaboró un diagrama de flujo con la descripción del método de obstrucción de los catéteres venosos con sangre, como se muestra en la Figura 1.

**Figura 1:** Descripción del método de obstrucción de los catéteres venosos centrales. Londrina, Paraná, 2022.

Autores de la investigación, 2022.

Los catéteres que no se obstruyeron se clasificaron como pérdidas y los primeros 15 catéteres ocluidos se incluyeron en la investigación. Si había más de 15 PICC obstruidos, se descartaban como pérdida, de modo que cada día cada grupo recibiera cinco catéteres de forma equitativa. La investigación contó con cuatro catéteres que no se obstruyeron y ocho fueron descartados para que hubiera homogeneidad entre los grupos.

Los primeros 15 dispositivos obstruidos se distribuyeron aleatoriamente mediante sorteo en tres grupos. Cada grupo recibió una solución diferente para probar su capacidad de desobstrucción, la distribución de los números de los grupos se realizó de forma aleatoria mediante sorteo.

El grupo 1 recibió una jeringa de 10 ml llena solo con solución salina.

El grupo 2 recibió vitamina C inyectable en una concentración de 25 mg/ml diluida en solución salina en una jeringa de 10 ml. Debido a la ausencia de estudios sobre el uso de vitamina C inyectable en la desobstrucción de catéteres en recién nacidos, la concentración de la solución se calculó basándose en la dosis diaria recomendada para bebés prematuros, que es de 15–25 mg/kg/día^(18)^.

El grupo 3 recibió heparina de bajo peso molecular en una dosis de 50 mg/mL diluida en solución salina en una jeringa de 10 mL. La dosis de heparina de bajo peso molecular se determinó mediante un estudio experimental*in vitro*realizado previamente, que determinó que la heparina de bajo peso molecular en una concentración de 50 mg/mL era eficaz para desobstruir el PICC de los recién nacidos^(16)^.

Las jeringas con las soluciones se protegieron de la luz con papel laminado y se les colocó una cinta de color, cuyo color se determinó mediante sorteo para diferenciar la solución que se estaba probando. El suero fisiológico, grupo 1, recibió una cinta de color verde; la vitamina C, grupo 2, recibió una cinta de color amarillo; y la heparina, grupo 3, recibió una cinta de color rojo.

La técnica adoptada para desobstruir el PICC fue la presión negativa mediante un grifo de tres vías, conectando el catéter obstruido a una de las vías, en el extremo proximal del grifo se colocó una jeringa Luer-Lock de 10 ml vacía y en el extremo distal del grifo de tres vías se colocó una jeringa Luer-Lock de 10 ml con la solución en prueba^(19)^.

En esta técnica, se cierra la vía de la solución y se aspira el contenido del lumen del catéter hasta la marca de 10 ml de la jeringa vacía; a continuación, se cierra la vía de la jeringa vacía y se abre la vía de la jeringa con solución, lo que permite que la solución llene el catéter mediante la presión negativa generada en el lumen del PICC. En ningún momento se realiza la técnica de infusión, solo la aspiración, de esta forma, se busca aspirar los coágulos y la solución^(19)^.

El intento de desobstrucción se realizó con los catéteres sumergidos en un baño María con agua destilada a 37 °C durante 1, 5, 10, 15, 30, 60, 120 y 240 minutos, y en cada intervalo de tiempo se realizaron tres intentos de desobstrucción. Estos intervalos de tiempo se determinaron para un mejor análisis de la progresión aritmética. Cada 240 minutos, si no se recuperaba la permeabilidad del PICC, se preparaba una nueva solución, debido a la inestabilidad de la heparina. Se determinó un tiempo máximo de desobstrucción de 24 horas tras la identificación de la oclusión.

Se consideró que se había recuperado la permeabilidad del catéter venoso cuando se observó la aspiración de los coágulos y del agua destilada del baño María en el que estaban sumergidos. Para confirmar la desobstrucción, se infundió 1 ml de solución salina en un dispositivo graduado en el tiempo de un minuto. Tras el inicio de la recopilación de datos, no fue necesario realizar ningún cambio en el protocolo del estudio.

### Aleatorización

La aleatorización simple fue realizada por un profesional externo a la investigación, responsable de preparar tres sobres opacos, identificados con cintas de colores verde, azul y amarillo, que determinaban a qué grupo pertenecía cada solución. Este mismo profesional también identificó las jeringas con los colores y soluciones correspondientes. El orden de selección de los PICC para el intento de desobstrucción se determinó mediante aleatorización simple, como se muestra en el diagrama de flujo de la Figura 2.

**Figura 2:** Descripción de la aleatorización de los catéteres venosos centrales obstruidos para el inicio de los intentos de desobstrucción. Londrina, Paraná, 2022.

Autores de la investigación, 2022.

### Cegamiento

En la Recolección de datos participaron dos investigadores: el investigador 1 se encargó de intentar desobstruir todos los catéteres a lo largo de los seis días y el investigador 2 se encargó de aleatorizar las soluciones que se iban a probar en cada catéter. El cegamiento fue realizado por el investigador 1, que era quien aplicaba la técnica de presión negativa para desobstruir el PICC, y por el responsable de realizar el análisis estadístico de los datos.

En una hoja de cálculo se anotó el número del catéter, el número de la solución (1, 2, 3), el número de intentos y el tiempo de desobstrucción. Se realizó una doble entrada y una doble verificación de los datos.

### Análisis y Tratamiento de los Datos

Las pruebas se realizaron con el programa Statistical Package for the Social Sciences (SPSS (25.0)). El análisis descriptivo se presentó mediante la media y la desviación estándar (DE). Las variables del número de intentos y el tiempo de desobstrucción se evaluaron mediante el análisis de normalidad de los datos a través de la prueba de Shapiro-Wilk. Las variables presentaron una distribución anormal, por lo que la diferencia entre los grupos de intervención se evaluó mediante la prueba de Kruskal-Wallis. Se adoptó un nivel de significación del 5,0% y p ≤ 0,05.

### Aspectos Éticos

Este estudio forma parte del proyecto de investigación titulado «Barreras y potencialidades en el cuidado del catéter central de inserción periférica en neonatología: en busca de innovaciones». La investigación fue aprobada por el Comité de Ética en Investigación, con el n.º CEP/UEL: 4.324.095. CAAE: 38696720.9.0000.5231 y se llevó a cabo de acuerdo con los preceptos éticos necesarios.

Se dispensó la necesidad de obtener la aprobación de los padres y/o tutores de los recién nacidos mediante la firma del término de consentimiento libre e informado (TCLE) para el uso del PICC que se encontraba en su hijo, ya que el dispositivo venoso sería desechado, ya que su extracción se produjo después de finalizar el tratamiento. Además, al haberse realizado un*flush*con 2 ml de agua destilada, los catéteres no contenían material biológico del paciente.

## RESULTADOS

Se elaboró la Tabla 1, que contiene la caracterización de los 90 PICC que componían la muestra. Se observa que la longitud de almacenamiento más frecuente del catéter fue de 16 a 20 cm, la mayoría de los catéteres se almacenaron durante un periodo de 0 a 6 meses, de 7 a 12 días fue el periodo de permanencia en el recién nacido con mayor prevalencia, la mayoría de los dispositivos recibieron la infusión de antibioterapia y todos los catéteres se retiraron debido a la finalización del tratamiento.

**Tabla 1:** Caracterización de los PICC que componían la muestra – Londrina, PR, Brasil, 2022.

Características	Cateteres que componían la muestra (n = 90)
Longitud del catéter en almacenamiento	24,45% (11 a 15 cm)
	31,12% (16 a 20 cm)
	27,78% (21 a 25 cm)
	16,65% (26 a 30 cm)
Período de almacenamiento del catéter	31,12% (0 a 6 meses)
	18,88% (7 a 12 meses)
	27,77% (13 a 18 meses)
	22,23% (19 a 24 meses)
Tiempo de permanencia en el RN	62,06% (7 a 12 días)
	24,14% (13 a 18 días)
	6,90% (19 a 24 días)
	6,90% (25 a 30 días)
Tipo de terapia infundida	60,65% (antibioticoterapia)
	31,14% (nutrición parenteral)
	4,92% (droga vasoactiva)
	3,29% (solución glucosada con concentración de infusión de glucosa sobre 12)
Motivo de retiro	100% (término del tratamiento)

Fuente: Datos de la investigación.

En la Tabla 2 se observa que las medias y la desviación estándar en relación con los intentos de desobstrucción fueron de 8,47 (5,23) para el suero fisiológico (sustancia 1), 9,43 (5,61) de la vitamina C (sustancia 2), 8,20 (5,59) de la heparina (sustancia 3) y con un valor p de 0,639 según el*Kruskal-Wallis.*En cuanto al tiempo de desobstrucción en minutos, las medias y desviaciones estándar fueron de 30,80 (40,63) para el suero fisiológico (sustancia 1), 35,43 (45,68) para la vitamina C (sustancia 2), 29,17 (41,17) de la heparina (sustancia 3) y con un valor p de 0,758 según el*Kruskal-Wallis.*

**Tabla 2:** Evaluación de la diferencia entre los grupos de intervención asociada al número de intentos y al tiempo en minutos – Londrina, PR, Brasil, 2022.

Variables X* (DE)**	Total	Soluciones			p-valor^†^
		1^††^	2^†††^	3^††††^	
Tentativas de desobstrucción	8,70 (5,45)	8,47 (5,23)	9,43 (5,61)	8,20 (5,59)	0,639
Tiempo (minutos)	31,80 (42,16)	30,80 (40,63)	35,43 (45,68)	29,17 (41,17)	0,758

X*: media.

DE**: Desviación Estándar.

^†^Test de Kruskal-Wallis.

1^††^: suero fisiológico.

2^†††^: vitamina C.

3^††††^: heparina.

Fuente: Datos de la investigación.

La Tabla 3 presenta la descripción de las soluciones para desobstruir los PICC en relación con los intervalos de tiempo y los intentos. Se observa que la cantidad de catéteres desobstruidos por cada una de las tres soluciones fue similar a lo largo de cada periodo de tiempo (1, 5, 10, 15, 30, 60 y 120 minutos). El tiempo máximo de desobstrucción de los catéteres por las tres soluciones fue de 120 minutos, pero no fueron necesarios el segundo y tercer intento de desobstrucción en el tiempo de 120 minutos para la solución de suero fisiológico, a diferencia de las soluciones de vitamina C y heparina.

**Tabla 3:** Desobstrucción de catéteres ocluidos en laboratorio según las tres soluciones en relación con el tiempo y el intento – Londrina, PR, Brasil, 2022.

Tiempo	Tentativa	Solución 1 (soro fisiológico)	Solución 2 (vitamina C)	Solución 3 (heparina)
		Cantidad de catéteres desobstruidos (n)		
1 minuto	1ª	1	0	2
	2ª	2	2	2
	3ª	6	6	6
Subtotal		9/30 (30%)	8/30 (26,66%)	10/30 (33,33%)
5 minutos	1ª	1	0	1
	2ª	2	2	3
	3ª	1	0	1
Subtotal		13/30 (43,33%)	10/30 (33,33%)	15/30 (50%)
10 minutos	1ª	2	2	0
	2ª	1	2	1
	3ª	1	3	2
Subtotal		17/30 (56,66%)	17/30 (56,66%)	18/30 (60%)
15 minutos	1ª	0	1	0
	2ª	0	0	1
	3ª	1	0	1
Subtotal		18/30 (60%)	18/30 (60%)	20/30 (66,66%)
30 minutos	1ª	0	1	0
	2ª	1	0	1
	3ª	3	3	1
Subtotal		22/30 (73,33%)	22/30 (73,33%)	22/30 (73,33%)
60 minutos	1ª	1	0	1
	2ª	1	0	2
	3ª	2	2	1
Subtotal		26/30 (86,66%)	24/30 (80%)	26/30 (86,66%)
120 minutos	1ª	4	0	1
	2ª	–	2	1
	3ª	–	4	2
Total		30/30 (100%)	30/30 (100%)	0/30 (100%)

Fuente: Datos de la investigación.

Durante los intentos de desobstrucción, no se observó a simple vista la presencia de posibles daños en la integridad del PICC ni el desprendimiento de coágulos visibles en su parte distal, pero fue posible visualizar la aspiración del coágulo hacia el interior de la jeringa en la que se aplicó la presión negativa.

## DISCUSIÓN

Al evaluar la eficacia de las soluciones de Suero fisiológico, heparina y vitamina C en la desobstrucción de catéteres venosos centrales neonatales ocluidos por coágulos, se observó que las tres sustancias fueron eficaces para desobstruir los catéteres por coágulos.

La obstrucción por coágulos dentro de la luz del PICC puede ser parcial o total, causada por la presencia de fibrina o trombos sanguíneos, lo que dificulta o imposibilita la administración de medicamentos debido a la falta de permeabilidad. Por lo general, se asocia con el flujo retrógrado dentro del catéter o con un fallo en la realización del*flushing* ^(20)^. El trombo es una de las principales causas de obstrucción de los dispositivos venosos centrales. La formación del trombo se produce por la llamada tríada de Virchow: estasis sanguínea, lesión del endotelio e hipercoagulabilidad^(21)^.

Esta investigación demostró que el suero fisiológico fue tan eficaz como la heparina en una concentración de 50 mg/mL y la vitamina C en una concentración de 25 mg/mL para desobstruir los PICC coagulados por sangre in vitro mediante el uso de la técnica de presión negativa.

El suero fisiológico está compuesto por una solución acuosa con una concentración del 0,9% de cloruro de sodio y puede utilizarse por vía intravenosa, intraocular, inhalatoria, entre otras. Es un compuesto con alta estabilidad tanto desde el punto de vista microbiológico como físico-químico^(22)^. Entre los cristaloides, el suero fisiológico al 0,9% es el más utilizado, a pesar de tener concentraciones elevadas de cloro y sodio en relación con el plasma. Tiene una osmolaridad mayor, pero cercana a la de la sangre. Tras la infusión de esta solución, el cloro y el sodio permanecen en el líquido extracelular, lo que permite considerar el suero fisiológico como una solución isotónica^(23)^. Se desconocen los efectos anticoagulantes del suero fisiológico.

La vitamina C es un micronutriente esencial que forma parte de varios procesos biológicos y bioquímicos. Hay dos presentaciones de la vitamina C en la sangre: el deshidroascorbato (forma oxidada) y el ácido ascórbico. Esta vitamina actúa bloqueando la adhesión de las plaquetas al endotelio vascular y mediante la agregación plaquetaria mediada por la P-selectina e inducida por la trombina^(24)^.

Corroborando los hallazgos de este estudio, investigaciones aleatorias que comparan la vitamina C y el Suero fisiológico han demostrado que no hay diferencias significativas entre estas dos soluciones en la prevención de la oclusión de catéteres venosos centrales^(12,25)^.

Ante coágulos pequeños, la infusión de ácido ascórbico desobstruye el vaso sanguíneo y elimina la adhesión de las plaquetas al endotelio. Además, la vitamina C actúa inhibiendo la liberación provocada por la trombina, que depende del pH del activador del inhibidor del plasminógeno-1 de las plaquetas. También reduce las concentraciones del factor de Willebrand y del activador del plasminógeno tisular, lo que demuestra su importante papel en la coagulación y la inflamación^(24)^. No hay pruebas científicas que aborden los efectos adversos de la vitamina C en la población neonatal.

La heparina está formada por cadenas de polisacáridos de varios pesos moleculares y tiene una alta afinidad con el inhibidor fisiológico de la coagulación, la antitrombina, el factor X activado (Xa) y la trombina (IIa). En este estudio se utilizó heparina de bajo peso molecular, cuya función anticoagulante se realiza a través de la antitrombina III, que actúa inhibiendo los factores de coagulación IXa, XIa, en menor medida, y XIIa, IIa y Xa en mayor intensidad^(26)^. La enoxaparina es la heparina de bajo peso molecular más utilizada, ya que necesita una dosis menor para que Xa alcance los valores de un adulto y tiene una depuración menor en los recién nacidos. Entre los efectos adversos, debe destacarse la hemorragia^(27)^.

Corroborando los hallazgos de este estudio, estudios aleatorios analizaron que la heparina y el suero fisiológico no presentaron diferencias estadísticas significativas en relación con la eficacia en el mantenimiento de la permeabilidad de los dispositivos venosos centrales, por lo que se recomendó el uso de solución salina^(28,29)^.

En cuanto a la desobstrucción de dispositivos venosos obstruidos por coágulos, se recomienda el uso de heparina, principalmente en el contexto nacional^(16)^, y de alteplasa, especialmente en el contexto internacional^(14)^. Hasta 1999, en Estados Unidos se recomendaba el uso de uroquinasa, pero la Administración de Alimentos y Medicamentos (FDA) prohibió el uso de este fármaco debido a los riesgos para su seguridad y eficacia, ya que se asoció a episodios de hemorragia grave^(14,30)^.

Es importante tener en cuenta que en los recién nacidos enfermos existe un desequilibrio en los niveles de estos factores de coagulación que puede estar relacionado con varias causas diferentes, como el estado procoagulante, el daño endotelial y la reducción del flujo, lo que favorece la formación de trombos o la aparición de hemorragias^(27)^.

Este estudio utilizó la técnica de presión negativa, sin embargo, en la literatura se encuentran dos técnicas para desobstruir catéteres centrales: la infusión de soluciones y la técnica de presión negativa^(31)^.

Cuando se produce una oclusión parcial del PICC, detectada por un aumento de la resistencia en la administración de medicamentos, se recomienda la técnica de infusión de la medicación con la solución para recuperar la permeabilidad. En caso de oclusión total del PICC, se debe utilizar la técnica de presión negativa para minimizar el riesgo de daños^(31)^.

Dado que la obstrucción puede producirse en cualquier segmento interno del PICC, es posible que el catéter contenga solución entre la obstrucción y el extremo proximal, por lo que la técnica de presión negativa tiene como objetivo retirar esta solución y provocar una presión negativa intraluminal en el dispositivo venoso. Cuando se libera la presión negativa, se aspira la sustancia presente en la otra jeringa que está conectada al catéter mediante la llave de tres vías^(32)^.

Esta técnica reduce los daños que la sobre-presurización puede provocar en el PICC y permite que la solución responsable de desobstruir el catéter llegue más rápidamente al trombo causante de la oclusión, ya que no es necesario forzar el émbolo de la jeringa para que la solución entre en la región intraluminal del dispositivo venoso^(32)^. La manipulación del catéter debe realizarse con una jeringa de al menos 10 ml para no provocar una presión superior a 25 psi dentro del catéter, evitando así su rotura y la aparición de daños en la red venosa^(33)^.

Se sabe que la obstrucción de un catéter venoso tiene una serie de consecuencias e impactos negativos para el recién nacido y su familia, entre ellos, la interrupción de la terapia farmacológica, el aumento de los costos hospitalarios, el estrés, el aumento de los procedimientos dolorosos y las inserciones repetidas para un nuevo dispositivo venoso^(34)^. Cabe destacar que el PICC en recién nacidos tiene mayores posibilidades de oclusión debido a su pequeño calibre^(12)^.

Los ensayos clínicos aleatorios demuestran la ausencia de evidencia científica que garantice la recomendación de utilizar heparina^(35)^, suero fisiológico y ácido ascórbico^(12)^para mantener la permeabilidad del catéter venoso central. Esto corrobora los hallazgos de esta investigación, que indican que no hubo diferencias estadísticas significativas entre las soluciones para desobstruir los PICC.

Se han desarrollado diversas estrategias con el objetivo de prevenir la obstrucción de los catéteres venosos, como la creación de una válvula antirreflujo que se coloca en el extremo distal del PICC para evitar el reflujo de sangre hacia el lumen del catéter. sin embargo, los estudios científicos no han demostrado una reducción en la tasa de oclusión y, por el contrario, es una estrategia que eleva el costo del dispositivo venoso^(36)^.

En cuanto al cuidado de los catéteres venosos, destaca el equipo de enfermería, principalmente el enfermero, que es el profesional responsable de definir y realizar el vendaje más adecuado para el paciente, además de tomar medidas para prevenir infecciones y obstrucciones. El enfermero es responsable de garantizar el cuidado en la manipulación, evaluar diariamente los dispositivos venosos y capacitar al equipo de salud responsable del cuidado del recién nacido^(37)^.

Entre los cuidados que deben realizarse para prevenir la oclusión se encuentra la instilación de Suero fisiológico con presión positiva para evitar que la sangre regrese al lumen del catéter^(19)^, lo que debe hacerse cada seis horas o antes y después de la infusión de medicamentos. Para ello, es necesario realizar la infusión de Suero fisiológico mediante la técnica pulsátil, con el fin de provocar un remolino en el interior del catéter para eliminar los depósitos de fibrina^(12)^.

Se considera una limitación el hecho de que no se hayan realizado análisis microscópicos del interior de los catéteres con el fin de verificar la desobstrucción parcial o total, por lo que la comprobación de la desobstrucción se realizó únicamente mediante examen clínico, es decir, se consideró desobstruido el catéter en el que, al instilar solución salina en la región proximal, se observó el fluido en su parte distal sin que se produjera resistencia a su infusión. También puede considerarse como un factor limitante los dos años de recolección y almacenamiento que fueron necesarios para alcanzar la cantidad suficiente de PICC para alcanzar el valor muestral, ya que, en ese tiempo, en la unidad neonatal a la que está vinculada esta investigación, hubo catéteres que fueron retirados por obstrucción y ruptura y que, por lo tanto, no pudieron ser incluidos en esta investigación.

Los estudios*in vitro*son esenciales para investigar los mecanismos biológicos y probar inicialmente la eficacia y seguridad de los procedimientos en un entorno controlado. Sin embargo, la validez de estos estudios para su aplicación directa en humanos, especialmente en neonatología, es limitada, ya que no reproducen íntegramente la complejidad del organismo vivo, incluidas las respuestas inmunológicas, el metabolismo y las interacciones celulares sistémicas. Por lo tanto, los hallazgos in vitro deben interpretarse como un paso preliminar que necesita confirmación mediante estudios in vivo y clínicos, garantizando la seguridad y eficacia reales para los pacientes neonatos^(38–40)^.

Además, la extrapolación directa de los resultados in vitro a la práctica clínica requiere precaución, ya que factores como el entorno fisiológico, la variabilidad individual y las condiciones clínicas específicas de los recién nacidos pueden influir en el comportamiento del dispositivo o la sustancia probada. Por lo tanto, para que las intervenciones basadas en datos*in vitro*se adopten en la atención de enfermería neonatal, es imprescindible realizar investigaciones clínicas rigurosas que validen estos hallazgos y promuevan la práctica basada en la evidencia, garantizando la calidad y la seguridad de la atención^(38,39)^.

Se espera que esta investigación sirva de base para futuros estudios que exploren alternativas más seguras y eficaces para la desobstrucción de PICC en recién nacidos, especialmente en lo que respecta a la comparación entre diferentes soluciones y técnicas utilizadas en la práctica clínica. Entre las principales lagunas que deben abordarse en futuras investigaciones destacan la evaluación de los efectos adversos a largo plazo, la estandarización de los protocolos de manejo y el análisis de la relación costo-beneficio de las intervenciones. Desde un punto de vista práctico, los resultados de este estudio pueden servir de base para la asistencia de enfermería, promoviendo conductas más seguras y basadas en la evidencia, lo que contribuye a la reducción de complicaciones y a la mejora de los resultados clínicos de los recién nacidos. Cabe destacar, además, la importancia de adoptar prácticas en salud basadas en la evidencia científica, que garanticen una atención cualificada y centrada en las necesidades de los pacientes.

## CONCLUSIÓN

Las tres sustancias utilizadas demostraron su eficacia para desobstruir los catéteres por coágulos, ya que todos los catéteres recuperaron su permeabilidad en un tiempo máximo de 120 minutos, mediante la técnica de presión negativa.

Este tiempo de hasta 120 minutos es viable en la práctica clínica, ya que el protocolo de desobstrucción de catéteres venosos centrales de la unidad neonatal, vinculada a este estudio, considera un tiempo máximo de 24 horas para la recuperación de la permeabilidad de estos dispositivos.

De este modo, la hipótesis de este estudio fue refutada, ya que no hubo significación estadística entre las soluciones utilizadas para desobstruir los PICC en relación con el tiempo y el número de intentos.

Se recomienda el uso de suero fisiológico, ya que posee estabilidad físico-química y microbiana, lo que conlleva menores efectos secundarios en relación con su uso en recién nacidos, especialmente en comparación con otras soluciones.

## Data Availability

Todo el conjunto de datos que apoya los resultados de este estudio fue publicado en el propio artículo.
